# Multimodal NIRS–MRI reveals cortical hypoperfusion, hypoxia, and mitochondrial changes in a mouse model of autoimmune inflammatory diseases

**DOI:** 10.1162/IMAG.a.1192

**Published:** 2026-04-02

**Authors:** Mada Hashem, Abbey Palset, Ying Wu, A. Max Hamilton, Emily C. Wuerch, V. Wee Yong, Jeff F. Dunn

**Affiliations:** Department of Radiology, Cumming School of Medicine, University of Calgary, Alberta, Canada; Hotchkiss Brain Institute, Cumming School of Medicine, University of Calgary, Alberta, Canada; Experimental Imaging Centre, Cumming School of Medicine, University of Calgary, Alberta, Canada; Department of Clinical Neurosciences, Cumming School of Medicine, University of Calgary, Alberta, Canada

**Keywords:** multimodal NIRS–MRI brain imaging, multiple sclerosis, neuroinflammatory diseases, cerebral blood flow, hypoxia, mitochondria

## Abstract

Abnormal oxidative metabolism and tissue hypoxia could exacerbate multiple sclerosis. Research in both animal models and people with multiple sclerosis shows increased inflammation, reduced cerebral blood flow, damage to mitochondria, and loss of myelin. Understanding such abnormalities is crucial for developing effective treatments. In this study, we apply a multimodal imaging approach—combining near-infrared spectroscopy (NIRS) with 9.4T MRI—to investigate the cortical gray matter of the experimental autoimmune encephalomyelitis (EAE) mouse model of autoimmune inflammatory diseases. Female C57BL/6J mice (*n* = 42) were used. EAE mice (*n* = 13) were induced using MOG_35-55_ peptide emulsified in complete Freund’s adjuvant (CFA) and pertussis toxin (PTX). Control groups were naïve (*n* = 15, no interventions), and CFA/PTX mice (*n* = 14, given CFA and PTX injections). We used NIRS–MRI to simultaneously monitor cerebral oxygenation, mitochondrial function (cytochrome c oxidase content and oxidation state), cerebral blood flow, and metabolic rate for oxygen consumption in the mice cortex at approximately peak disease. Both CFA/PTX and EAE groups showed reduced perfusion and tissue oxygenation (hypoxia), while the metabolic rate of oxygen did not change. The concentration of cytochrome c oxidase was lower with a higher oxidation state in EAE mice than naïve and CFA/PTX groups. Histology showed cortical gray matter microgliosis, but no obvious neuronal death or demyelination in EAE at peak disease. As reduced blood flow, hypoxia, and high oxidation state were observed in both CFA/PTX and EAE, it is possible that inflammation is causing these changes. Mitochondrial dysfunction appears in EAE mice, but increased oxygen extraction fraction and oxidation of cytochrome c oxidase compensate, allowing no change in the metabolic rate of oxygen consumption. Inflammation, damaged mitochondria, hypoxia, and inefficient energy production could exacerbate gray matter pathology in multiple sclerosis. By revealing cortical disruptions in oxygen delivery and consumption, the multimodal NIRS–MRI approach provides a powerful imaging tool for identifying potential biomarkers of disease physiology and progression.

## 1 Introduction

Multiple sclerosis (MS) is characterized primarily by demyelination, degeneration, and inflammation of the central nervous system (CNS) (Baecher-Allan et al., 2018). While MS is typically considered a white matter (WM) disease, there is prominent evidence that gray matter (GM) pathophysiology is involved as well (Davies et al., 2013; Haider et al., 2014; Yang & Dunn, 2019). Even regions of GM that appear to be relatively normal on conventional MRI—known as normal-appearing GM (NAGM)—may still experience significant physiological changes (Klaver et al., 2013). Research in both animal models and humans reveals increased inflammation, reduced cerebral blood flow (CBF), mitochondrial damage, and myelin loss (Geurts & Barkhof, 2008). We are interested in exploring changes in CBF, oxygen metabolic rate, and mitochondrial function in NAGM using a mouse model of MS.

The impact of demyelination on oxygen metabolism is an open question. Following demyelination, energy demand in the CNS could increase and lead to increased cerebral metabolic rate for oxygen (CMRO_2_) consumption (Ames III, 2000; Schiepers et al., 1997). When myelin is damaged or lost, axonal conduction becomes less efficient, leading to compensatory increases in neural activity that raise energy demand for maintaining ion gradients across cell membranes (Gerevich et al., 2023). Moreover, the repair and remyelination processes of damaged axons by glial cells require considerable metabolic activity, including increased oxygen uptake, and energy production (Alizadeh et al., 2015). Conversely, quantification of CMRO_2_ in the demyelinating cuprizone mouse model showed a reduction in CMRO_2_ (Hashem et al., 2022), or hypometabolism. Cerebral hypometabolism has been also associated with CNS degeneration and overall atrophy (Schiepers et al., 1997; West et al., 2020). However, this phenomenon is not well correlated with either atrophy or lesions (Ge et al., 2012; Sun et al., 1998). It has been suggested that neuroinflammation and neurodegeneration could be potentially linked by mitochondrial damage (Witte et al., 2010), emphasizing the critical importance of further research to understand the mechanisms underlying these disruptions.

Here we combine MRI with near-infrared spectroscopy (NIRS) to investigate oxidative metabolism and the role of mitochondria in the cortical GM of the inflammation-induced autoimmunity mouse model—Experimental Autoimmune Encephalomyelitis (EAE).

EAE is widely used to model MS and is characterized by infiltration of immune cells into the CNS, activation of microglia, and demyelination of axons. Historically, MS research has focused on spinal cord pathology and motor dysfunction in the EAE model (Davies et al., 2013; Desai et al., 2020; Nikić et al., 2011; Sadeghian et al., 2016; Tai et al., 2023). However, in humans, MS affects the brain in addition to the spinal cord, with observed neurodegeneration in the cortex and other areas of the brain (Trapp et al., 2018). Recent studies have established that EAE affects the cortical GM with elevated inflammatory markers (Acharjee et al., 2013; Chanaday & Roth, 2016) and pathology that progresses to neural degeneration in the cerebral cortex (Hamilton et al., 2019; MacKenzie-Graham et al., 2012). Studying the cerebral cortex pathology in the EAE model, and its underlying mechanisms, is increasingly important, as it addresses brain involvement in the disease and can potentially unveil new therapeutic targets, lead to earlier diagnosis, and improve treatment approaches.

Reduced CBF and changes in mitochondrial function were reported in people with multiple sclerosis (pwMS) (D’haeseleer et al., 2015; Witte et al., 2010). Assuming there are parallels with the human condition, we hypothesize that there will be evidence for reduced CBF and changes in mitochondrial function (consistent with mitochondrial damage) in the EAE model. Following from our work implicating inflammation as being interactive with hypoxia (Soroush & Dunn, 2025; Yang & Dunn, 2019), we also hypothesize that there will be evidence of hypoxia in NAGM of the EAE model. Finally, we quantify CMRO_2_ as a marker of metabolic rate. This could increase, if myelin damage increases metabolic rate to overcome reduced conduction efficiency. It may also decrease if there is cell loss. As abnormal oxidative metabolism in GM could precede degeneration and exacerbate the pathology, it is important to understand the interactions between these variables.

We applied a novel multimodal technique that combines NIRS with 9.4T MRI to monitor multiple oxidative metabolism parameters in the cerebral cortex (Hashem et al., 2020). The EAE group was compared with two experimental control groups, one is naïve, with no interventions, and the second is injected with the complete Freund’s adjuvant (CFA), followed by pertussis toxin (PTX), without adding the peptide to trigger autoimmunity. The CFA/PTX group was included since adjuvants can stimulate an immune response. Female mice were used because EAE induction is more consistent and exhibits higher disease incidence and severity in females, thereby improving model reliability and reducing variability (McCombe & Greer, 2022). This also reflects the fact that in humans, there is a higher prevalence of MS in females, reported as high as a 3:1 female-to-male ratio (Feigin et al., 2019; Jakimovski et al., 2024). Control mice (naïve and CFA/PTX) were female to be consistent with the EAE model. NIRS detects hemoglobin (Hb) and the mitochondrial enzyme cytochrome c oxidase (CCO) that are both strongly linked to oxygenation and metabolism. Measuring the absolute concentration of oxygenated, deoxygenated, and total Hb allows the assessment of oxygen saturation in the tissue microvasculature (S_t_O_2_) as a measure of hypoxia (Zhang et al., 2010). Quantifying the oxidation state and the concentration of CCO determines whether mitochondrial function is affected. Cerebral blood flow (CBF), a measure of perfusion, was quantified with Arterial Spin Labeling (ASL) MRI. Oxygen extraction fraction (OEF) and CMRO_2_ were calculated using the modified Fick Principle (Kety & Schmidt, 1948), as a measure of oxygen metabolism and energy demand in the brain. This is the first study to combine NIRS with MRI to assess metrics of oxidative metabolism in the cortex, and to study the interaction of hypoxia, perfusion, metabolic rate, and mitochondrial function in the EAE mouse, as a model of MS.

## 2 Materials and Methods

### 2.1 Mice

Female C57BL/6J mice (*n* = 42) at 6–7 weeks of age were purchased from Jackson Laboratory, ME, US. The mice were housed and maintained in the University of Calgary Animal Care facility with a 12-hour light-and-dark cycle with access to water and food pellets *ad libitum*. Mice were allowed to acclimatize for 1 week. Animal protocols were approved by the Health Sciences Animal Care Committee (HSACC) of the University of Calgary and conformed to the guidelines established by the Canadian Council of Animal Care (CCAC). The experiments conducted in this study have been reported in compliance with the Animal Research: Reporting of In Vivo Experiments (ARRIVE) guidelines.

### 2.2 EAE induction

At 8–9 weeks old, mice were randomly separated into naïve (*n* = 15), CFA/PTX (*n* = 14), and EAE (*n* = 13) groups. Female mice were selected because of their greater susceptibility and more consistent induction of EAE (McCombe & Greer, 2022). Control groups also consisted of female mice to avoid sex-related confounding across groups. EAE mice were immunized with 50 µg of myelin oligodendrocyte glycoprotein (MOG_35-55_; synthesized by protein and nucleic acid facility, Stanford University), emulsified with Complete Freund’s Adjuvant (CFA; Thermo Fisher Scientific), and 10 mg/ml of heat-inactivated *mycobacterium tuberculosis* (Sigma-Aldrich). Using CFA in mice elicits consistent EAE and creates a proinflammatory environment of cytokines leading to a severe course of disease. Mice were lightly anesthetized with ketamine/xylazine, and EAE was induced via one 50 μl subcutaneous injection into each hind flank (50 µg of MOG/mouse total). In addition, EAE mice were intraperitoneally injected with 300 ng of pertussis toxin (PTX; List Biological Laboratories) in 200 μl volume immediately and 2 days after MOG immunization.

To assess disease severity, EAE mice were scored daily on a 15-point grading scale, summing the condition of the tail and each of the 4 limbs (Weaver et al., 2005). For the tail, 0 signifies no signs, 1 indicates half-paralysis, and a maximum score of 2 is given for complete paralysis. For each of the limbs, 0 is given for no signs, 1 for weak or altered gait, 2 for paresis, and a maximum score of 3 for a fully paralyzed limb. A total score of 15 indicates death. CFA/PTX control mice received all injections similar to EAE mice, but CFA was emulsified in PBS instead of MOG. Naïve mice received no injections. The three mouse groups were subjected to the same handling and behavior scoring. Imaging was scheduled at peak disease, defined as the day when mice reached a behavior score of 10, or when they reached 17 days post-induction (which is past peak disease based on our previous studies). CFA/PTX and naïve mice were sacrificed in a pattern that matched EAE. The main experimenter was blinded to the group assignments and scoring process throughout the experiment, including during data collection and analysis.

### 2.3 NIRS–MR imaging

The experimental setup of the multimodal NIRS–MRI system was described previously (Hashem et al., 2020, 2021, 2022). The system consists of a 9.4 T horizontal bore MRI (Bruker Avance console, Bruker Biospin GmbH, Rheinstetten, Germany) with a 35 mm quadrature volume coil, and a custom-built continuous-wave broadband NIRS system, which were operated by two independent computers. The NIRS system incorporated a broadband white light source (77501; Oriel Instruments Inc., USA), two Hard-Clad Silica Core Multimode optical fibers (FT1000EMT; Thorlabs Inc, USA) with a 1000 µm core diameter and 0.39 NA, a spectrograph (Shamrock 303i; Andor Technology Inc, Northern Ireland), and a CCD camera (iDus 420; Andor Technology Inc, Northern Ireland). A GRIN lens with a 1.80 mm diameter and 0.55 NA (#64-525; Edmund Optics, USA) and a 90˚prism with a leg length of 2 mm (#45-524; TECHSPEC NSF11, Edmund Optics, USA) were glued to the end of the fibers to collimate the light and direct it from the source fiber into the tissue and from the tissue into the detector fiber. NIR attenuation spectra were collected continuously from the mouse cortex at a sampling rate of 6 Hz over the range of 705–960 nm.

Mice were anesthetized with 5% isoflurane added to a gas mixture of 70% N_2_ and 30% O_2_. The optic fibers (one source and one detector) were secured on top of the shaved head of the mouse, around the bregma (±1 mm), by covering the area with masking tape connected to the outside of the MRI coil cradle. Fibers were separated with a black rubber separator to maintain the optimal distance (4 mm) between the source and detector fibers and to reduce direct illumination between them. This separation distance was determined by simulations of light propagation and ensures that the optical signal emanates primarily from the cerebral cortex GM (Johnson, 2017). Light coupling to the skin was enhanced by covering the prisms with a thin layer of glycerol (Bashkatov et al., 2002). A small water phantom was mounted on top of the prisms to facilitate selection of the MR slice corresponding to the brain area measured with NIRS. Heart rate, breathing rate, and arterial oxygen saturation (S_a_O_2_) were monitored by the MouseOx MRI-compatible pulse oximeter (Starr Life Sciences, USA). The mouse with the optic fibers on the head and the pulse oximeter on the thigh was placed on a heating pad and secured in the MRI coil. Core body temperature was maintained at 36.5 ± 0.1˚C during data acquisition. Mice were monitored for 10 minutes prior to data acquisition, to ensure stable physiology.

Arterial Spin Labeling (ASL) MRI was applied to measure perfusion. A single axial slice was acquired around bregma (±1mm), featuring the frontal lobe and some of the parietal lobe, using a CASL-HASTE sequence with TR = 3000 ms, TE = 13.5 ms, FOV = 25.6 × 25.6 mm, matrix size = 128 × 128 pixels, slice thickness = 1.5 mm, 16 averages. Four perfusion images (two control and two tagged) were collected per measurement to correct for magnetization transfer. A T1 map was obtained in the same location using a RARE-VTR sequence where effective TE = 20 ms, TR = 100, 500, 1000, 3000, and 7500 ms. Together, the four perfusion images and the T1 map were collected over a period of 14 minutes.

During data acquisition, anesthesia was maintained at 1.7%–2% isoflurane. NIRS and pulse oximeter data were collected continuously over the imaging period, which lasted 25 minutes. To assess the concentration of hemoglobin, an anoxia pulse was given (50 seconds) in the last minute, where the air mixture ratio was altered to 100% N_2_/0% O_2_ (Zhang et al., 2010). After the anoxia pulse, the O_2_ level was restored to 30% for recovery. NIRS data were averaged over the time of the ASL acquisition period for the calculation of chromophore concentrations.

### 2.4 Data analysis

Individual perfusion maps were calculated for each animal (Hashem et al., 2020, 2022). An ROI from the cerebral cortex, estimated to be similar to the region of sensitivity for NIRS, and covering primarily the motor and the somatosensory area, was created using a tagged image (better contrast). This ROI was then used on the perfusion maps to obtain a mean ± SD (ml ⋅ 100g^-1^ ⋅ min^-1^) for perfusion. A second individual drew the ROIs in a blinded fashion and the results for each were compared. If the difference was over 5%, the analysis was repeated. If not, the data from the primary analysis were used.

The concentrations of the main chromophores in the tissue: deoxyhemoglobin (dHb), oxidized CCO (oxCCO), and reduced CCO (reCCO) were quantified from the measured attenuation light using the NIR-AQUA algorithm described in our previous studies (Hashem et al., 2020, 2021; Zhang et al., 2010). The main assumption underlying this algorithm is that tissue scattering and water content remain constant throughout the measurement period. Therefore, NIR-AQUA is based on the second derivative approach, commonly applied in broadband NIRS methods (Matcher & Cooper, 1994; Matcher et al., 1994), to remove the scattering effect. It also uses the absorption features of water (at 800–850 nm) and its known concentration in the brain tissue of rodents (80%) to estimate the mean optical pathlength that the photons travel. The algorithm applies multilinear regression to fit the measured attenuation spectra to the specific absorption coefficients of each chromophore, downloaded from the University College of London medical physics website, over a specific wavelength range for each chromophore (UCL, 2005). The total concentration of CCO (totCCO) is the sum of oxCCO and reCCO. The total concentration of hemoglobin (tHb) in the cerebral tissue was determined using the anoxia pulse method (Cooper et al., 1998; Zhang et al., 2010), in which it is assumed that during the anoxia pulse, tHb is approximated by the total dHb concentration ([tHb] = [dHb]).

Tissue oxygen saturation (S_t_O_2_), that is, the oxyhemoglobin saturation in the tissue microvasculature, was determined using dHb and tHb concentrations (Hashem et al., 2020). The oxygen extraction fraction (OEF) was calculated as the arteriovenous oxygen saturation difference divided by S_a_O_2_. Venous oxygen saturation (S_v_O_2_) was estimated from S_a_O_2_ and S_t_O_2_ values, where the arterial/venous Hb ratio used is 0.25/0.75 (Hashem et al., 2020; Tichauer et al., 2006). CMRO_2_ was quantified using the modified Fick principle (Hashem et al., 2022, 2023; Tichauer et al., 2006).

### 2.5 Histology

Following the imaging session, mice were perfusion fixed under 5% inspired isoflurane, using phosphate buffered saline (PBS; Thermo Fisher Scientific) followed by 4% paraformaldehyde (PFA; Sigma-Aldrich). Mouse brains were removed and placed in 4% PFA overnight before being placed in a 30% sucrose solution. Frozen sectioning was done using a cryostat, whereby tissue was cut into 20 μm thick slices for staining. Slices from naïve, CFA/PTX, and EAE mice were stored at -80˚C.

Fluoro-Jade C (FJC) kit (Biosensis, US) was used as a selective stain for degenerating neurons. Frozen sections were rehydrated, then incubated in basic alcohol for 5 minutes, followed by 0.06% KMnO_4_ for 10 minutes. The sections were then drained and rinsed for 2 minutes with distilled water, followed by incubation in FJC solution for 10 minutes. Following incubation, slides were rinsed three times in distilled water and dried for 1 hour in a dark environment.

Myelin basic protein (MBP) antibody (Abcam Cat# ab40390) was used as a marker for myelin assessment. Ionized calcium binding adaptor molecule 1 (Iba1; Wako Cat# 019-19741) was used to stain microglia and determine immune activation. Vectashield Vibrance antifade mounting medium with DAPI (MJS BIOLYNX INC, Cat# VECTH180010) was used to stain dsDNA from cell nuclei. Alexa Fluor 488 and 594 (Jackson ImmunoResearch Labs, Cat# 711-545-152, Cat# 711-585-152) were used as secondary antibodies. Prior to staining, slices were thawed and dried at room temperature for 30 minutes. Afterward, tissue was blocked for 1 hour with 10% goat serum and 1% Triton-X100 in PBS. Primary antibodies (MBP, Iba1) were diluted and incubated overnight with 1% goat serum and 1% Triton-X100 in PBS. After 24 hours of incubation, slices were washed in 1XPBS. Secondary antibodies were diluted and added to the slices with 1% goat serum and 1% Triton-X100 in PBS. After 1 hour of incubation, slices were washed in 1XPBS. DAPI mounting medium was added, and a coverslip was placed on top. Slides were then observed under a fluorescent microscope at 10X and 20X magnification. For each stain, six slices 300 µm apart were processed per mouse.

### 2.6 Stereology

Cerebral cortex sections were analyzed (*n* = 4 naïve, 4 CFA/PTX, 4 EAE). Six slices were used for each mouse. Microglia counting and cell size estimation were performed using Fiji (ImageJ) version 1.51b. For each tissue slice, three adjacent images were taken of each side of the cortex for a total of six images per slice. Counting was performed at 10X magnification (NA 0.24). The size of microglia was defined as the area of each stained cell. To avoid experimenter bias, threshold of staining signal was used, and stained particles outside the range 150–1000 pixels^2^ were excluded.

### 2.7 Statistical analysis

Sample size (*n*) was determined using a power analysis. One parameter that typically exhibits large variance is perfusion data. Based on the expected mean and variance at baseline in naïve mice, a difference of 20 ml ⋅ 100g^-1^ ⋅ min^-1^ (α = 0.05, power level: 95%) could be detected with a minimum of *n* = 4 per group. However, since in this study we measured and derived multiple metrics, including parameters with smaller expected differences, and to ensure sufficient statistical power across analyses while avoiding type I and type II errors, the sample size was increased to *n* = 13–15 per group. Slight variations in group sizes reflect exclusions due to technical artifacts or data quality issues.

A Mixed-Design Repeated Measures ANOVA with Bonferroni corrections was performed to determine when a significant difference in the mean disease score starts appearing between EAE and CFA/PTX groups over the 17-day period. Post hoc pairwise comparisons were conducted to evaluate within-group longitudinal changes in EAE over time.

Normality was assessed using the Shapiro–Wilk test. As most variables did not meet normality assumptions, the non-parametric Kruskal–Wallis test, including all pairwise comparisons with Bonferroni corrections for multiplicity, was conducted to determine significant changes in oxidative metabolism correlates between naïve, CFA/PTX, and EAE mouse groups. All data were expressed as mean ± SD, and *P* < 0.05 was considered statistically significant. Pearson’s correlation coefficient (r) was used to assess the strength and direction of the relationship between variables. Statistical significance for stereology data was determined using a repeated measure (RM) one-way ANOVA test, where *P* < 0.05 was considered statistically significant. All statistical analyses were performed in IBM SPSS Statistics v24.

## 3 Results

**Fig. 1.**
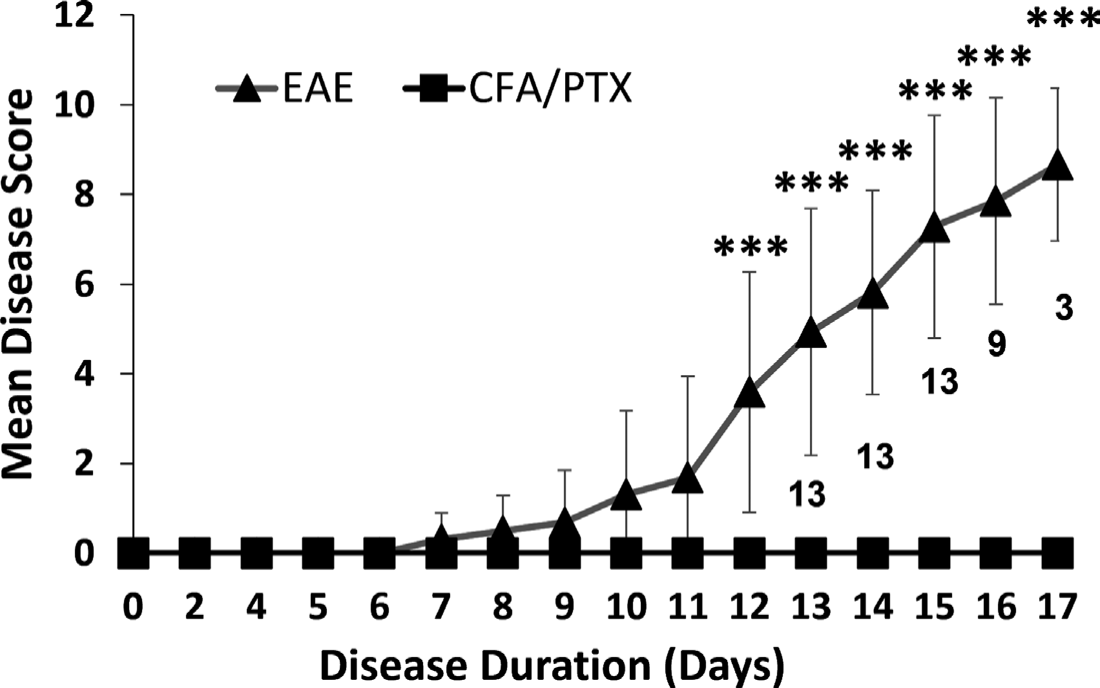
Disease course of EAE and CFA/PTX mice. Mean daily disease score (mean ± SD) of EAE (*n* = 13 on day 0, *n* = 3 on day 17) and CFA/PTX (*n* = 14 on day 0, *n* = 5 on day 17) mice following immunization. The mean disease score on days 15–17 post-induction was 0 for CFA/PTX and 7.9 ± 0.7 for EAE. Numbers on the graph represent the number of EAE mice left and scored on the corresponding day. Significant differences between EAE and CFA/PTX start on day 12 (*P* < 0.001). Statistics were performed using a Mixed-Design Repeated Measures ANOVA with Bonferroni corrections, ***P < 0.001.

EAE mice showed peak disease scores at days 15–17 post-immunization (Fig. 1). Significant differences between EAE and CFA/PTX emerged on day 12 (*P* < 0.001). Post hoc comparisons showed no significant changes between days 15 and 17 in the EAE group, indicating a plateau consistent with peak disease. Naïve, CFA/PTX, and EAE mice were imaged with the multimodal NIRS–MRI at days 15–17 post-EAE induction.

**Fig. 2.**
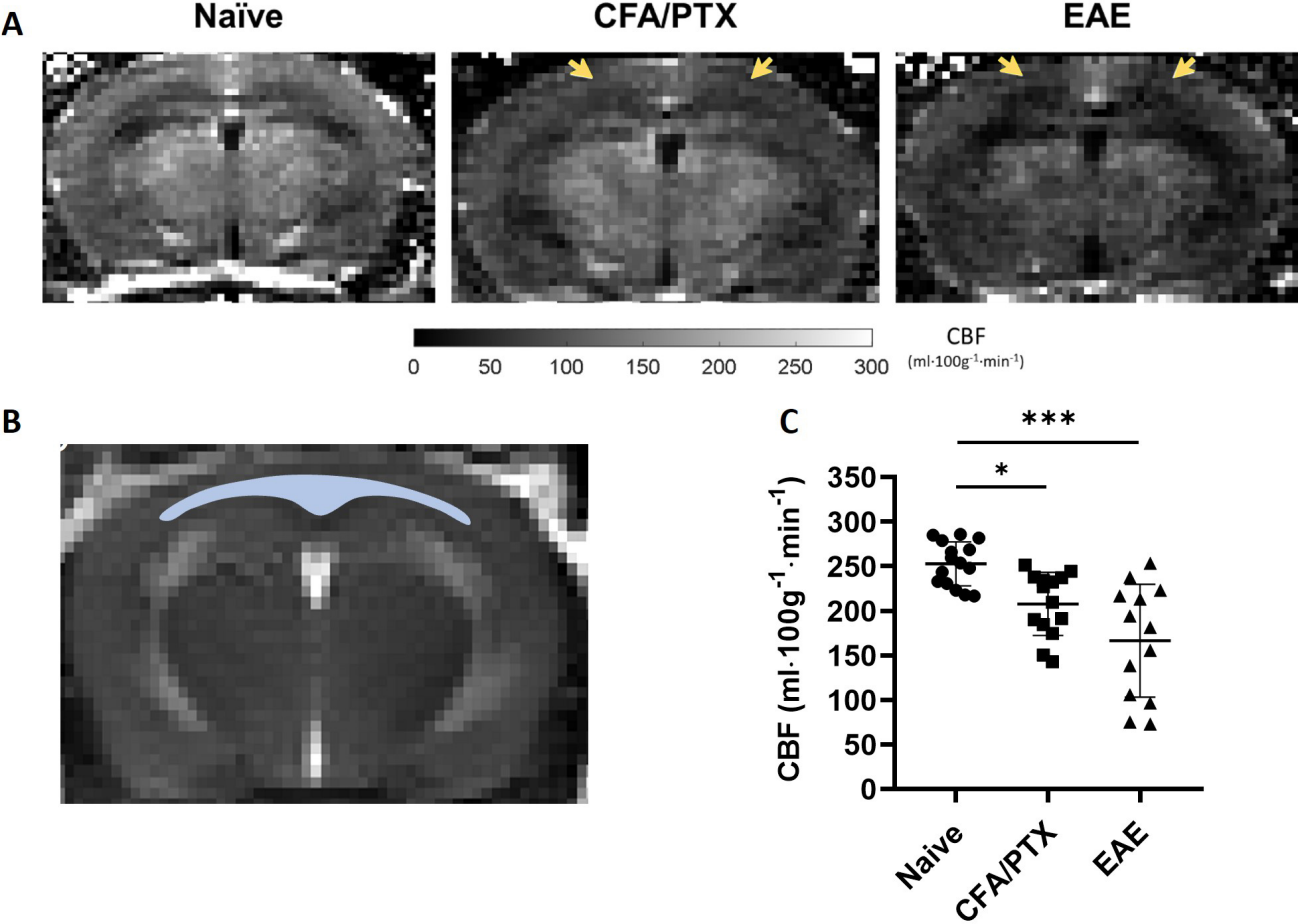
Cerebral blood flow (CBF) in the cortex. (A) Representative perfusion maps from a naïve, CFA/PTX, and EAE mouse at peak disease. Both CFA/PTX and EAE mice show clear hypoperfusion (yellow arrows) in the cerebral cortex. (B) ROI from cerebral cortex (blue), covering the motor and the somatosensory area, was selected on a tagged image and used to calculate average CBF in the cortex. (C) Reduced CBF was found in CFA/PTX and EAE mice at peak disease. Each symbol represents a different mouse (*n* = 15 naïve, 14 CFA/PTX, 13 EAE). The Kruskal–Wallis test with all pairwise comparisons and Bonferroni corrections was conducted to determine significant differences (*P < 0.05, ***P < 0.001).

Figure 2A shows representative perfusion maps from a naïve, a CFA/PTX, and an EAE mouse at peak disease. Hypoperfusion was clear, mainly in the cortex of CFA/PTX and EAE mice. Average CBF in the cortex was obtained from the perfusion maps using the ROI shown in Figure 2B, which was initially created on the respective tagged images. Figure 2C shows the quantified CBF in the cortex of the three mouse groups. There was a significant decrease in CFA/PTX (207.7 ± 35.5 ml ⋅ 100g^-1^ ⋅ min^-1^, *P* = 0.02) and EAE mice at peak disease (166.4 ± 63.3 ml ⋅ 100g^-1^ ⋅ min^-1^, *P* < 0.001), compared with the naïve group (252.6 ± 24.6 ml ⋅ 100g^-1^ ⋅ min^-1^). There was no significant difference in CBF between CFA/PTX and EAE mice (*P* = 0.5).

**Fig. 3.**
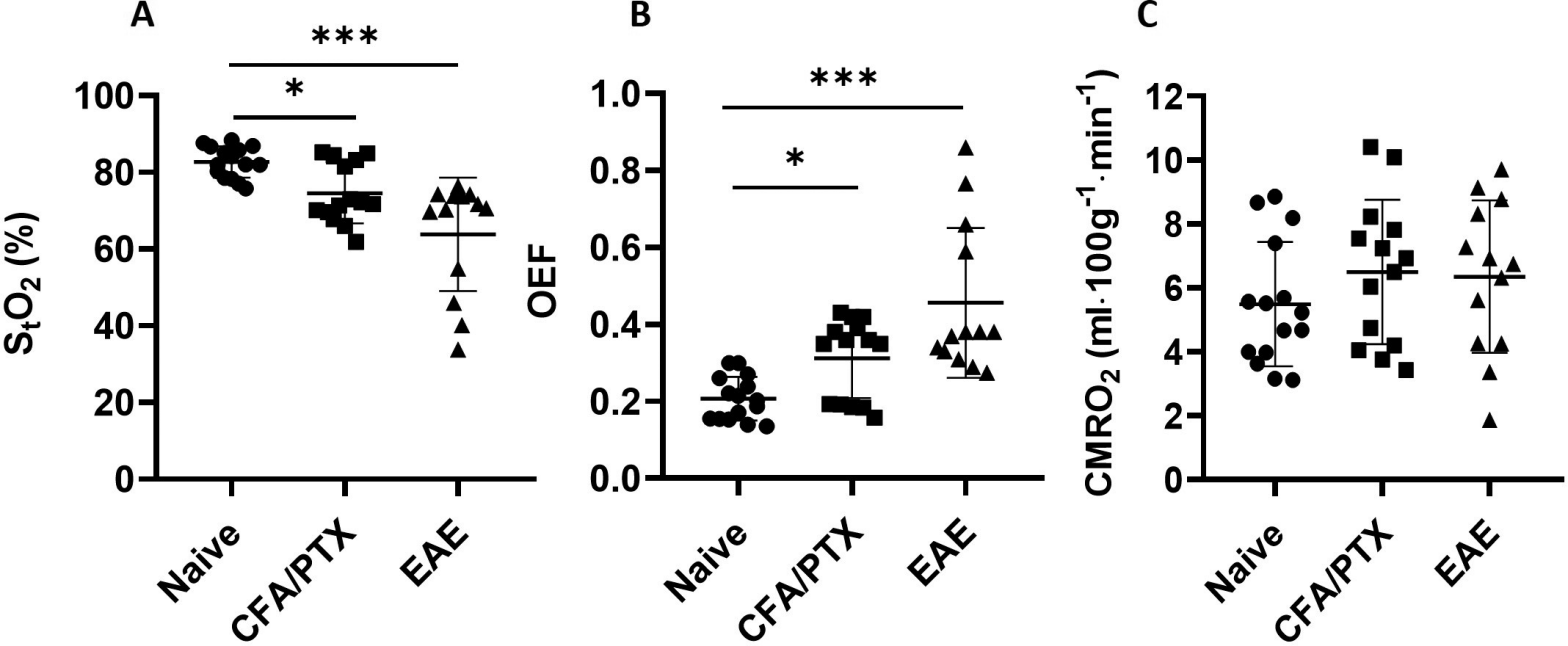
Oxidative metabolism metrics in the cortex. Reduced (A) tissue oxygen saturation (S_t_O_2_) and increased (B) oxygen extraction fraction (OEF) with no change in (C) cerebral metabolic rate for oxygen (CMRO_2_) in CFA/PTX and EAE mice. Each symbol represents a different mouse (*n* = 15 naïve, 14 CFA/PTX, 13 EAE). The Kruskal-Wallis test with all pairwise comparisons and Bonferroni corrections was conducted to determine significant differences (*P < 0.05, ***P < 0.001).

Both CFA/PTX and EAE mice showed cortical hypoxia, as measured with S_t_O_2_, compared with naïve mice. Figure 3A shows a significant decrease in S_t_O_2_ of CFA/PTX (74.5 ± 7.8%, *P* = 0.02) and EAE (63.8 ± 14.8%, *P* < 0.001) mice, compared with the naïve group (82.7 ± 4.1%). There was no significant difference in S_t_O_2_ between CFA/PTX and EAE mice (*P* = 0.4) at peak disease.

Figure 3B and 3C shows OEF and CMRO_2_. OEF increased significantly in CFA/PTX (0.31 ± 0.10, *P* = 0.02) and EAE (0.46 ± 0.19, *P* < 0.001) mice at peak disease, compared with the naïve group (0.21 ± 0.05). There was no significant difference in OEF between CFA/PTX and EAE mice *(P* = 0.4). CMRO_2_ did not differ significantly among the three mouse groups (*P* = 0.5), although slightly higher average values were observed in CFA/PTX (6.5 ± 2.2 ml ⋅ 100g^-1^ ⋅ min^-1^) and EAE mice (6.3 ± 2.4 ml ⋅ 100g^-1^ ⋅ min^-1^), compared with naïve mice (5.5 ± 1.9 ml ⋅ 100g^-1^  ⋅ min^-1^).

**Fig. 4.**
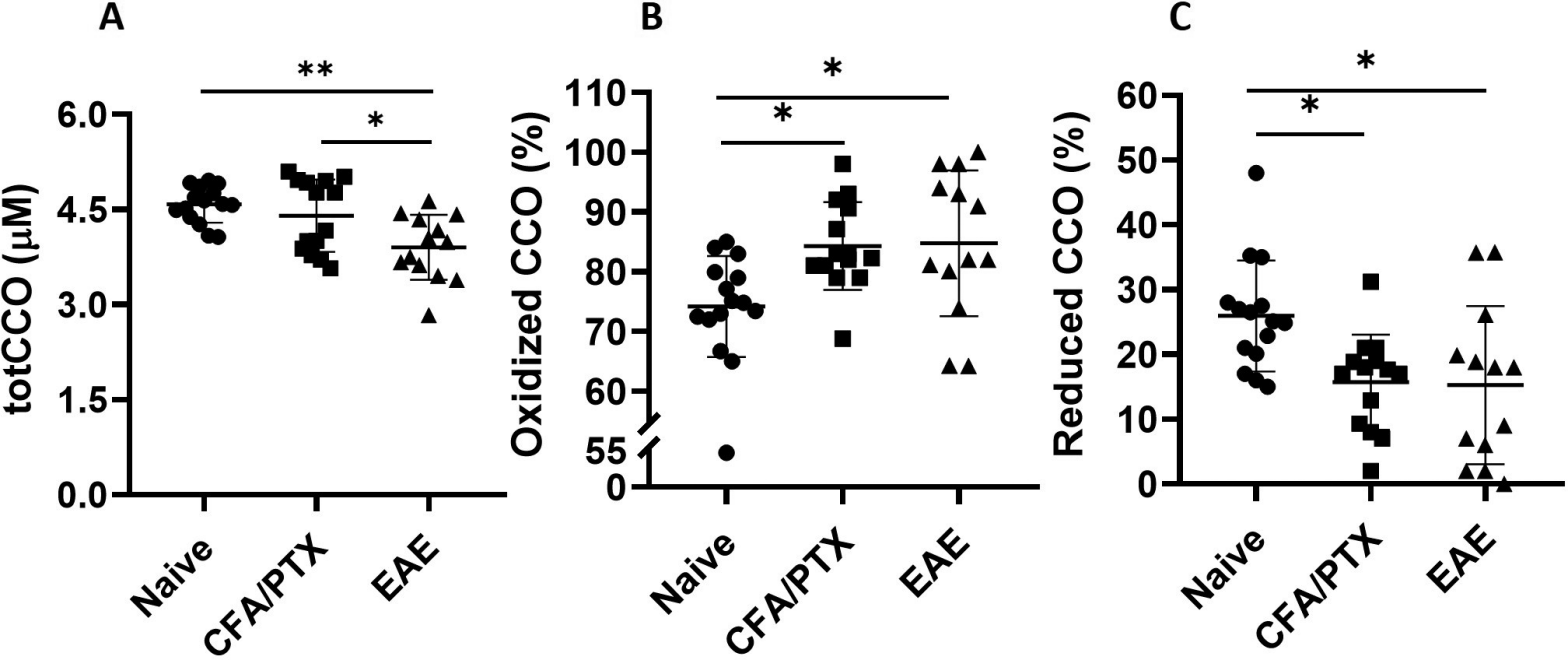
Cytochrome C Oxidase content and oxidation state as markers of mitochondrial function. Decreased cytochrome c oxidase (totCCO) content (A) and increased oxidation state 15–17 days following EAE induction. Oxidized (B) and Reduced (C) CCO (%) are the fraction of the enzyme that is oxidized or reduced, respectively. Each symbol represents a different mouse (*n* = 15 naïve, 14 CFA/PTX, 13 EAE). The Kruskal–Wallis test with all pairwise comparisons and Bonferroni corrections was conducted to determine significant differences (*P < 0.05, **P < 0.01).

Figure 4 shows the concentration and oxidation state of CCO in the cortex. There was a significant decrease in cortical CCO content (totCCO) in EAE mice at peak disease (3.9 ± 0.5 µM) compared with both naïve (4.6 ± 0.3 µM, *P* = 0.004) and CFA/PTX (4.4 ± 0.6 µM, *P* = 0.04) mice. CCO concentration was not significantly different between naïve and CFA/PTX mice (*P* = 1.0). Furthermore, 85 ± 1% of the enzyme in EAE was in its oxidized state, compared with 84 ± 1% in CFA/PTX (*P* = 1.0) and 74 ± 1% in naïve (*P* = 0.03) mice. The oxidation state of CCO in CFA/PTX mice was higher than in naïve mice (*P* = 0.03). The high variability in the EAE and CFA/PTX groups shows that some animals may not be exhibiting the same changes at a given time.

**Fig. 5.**
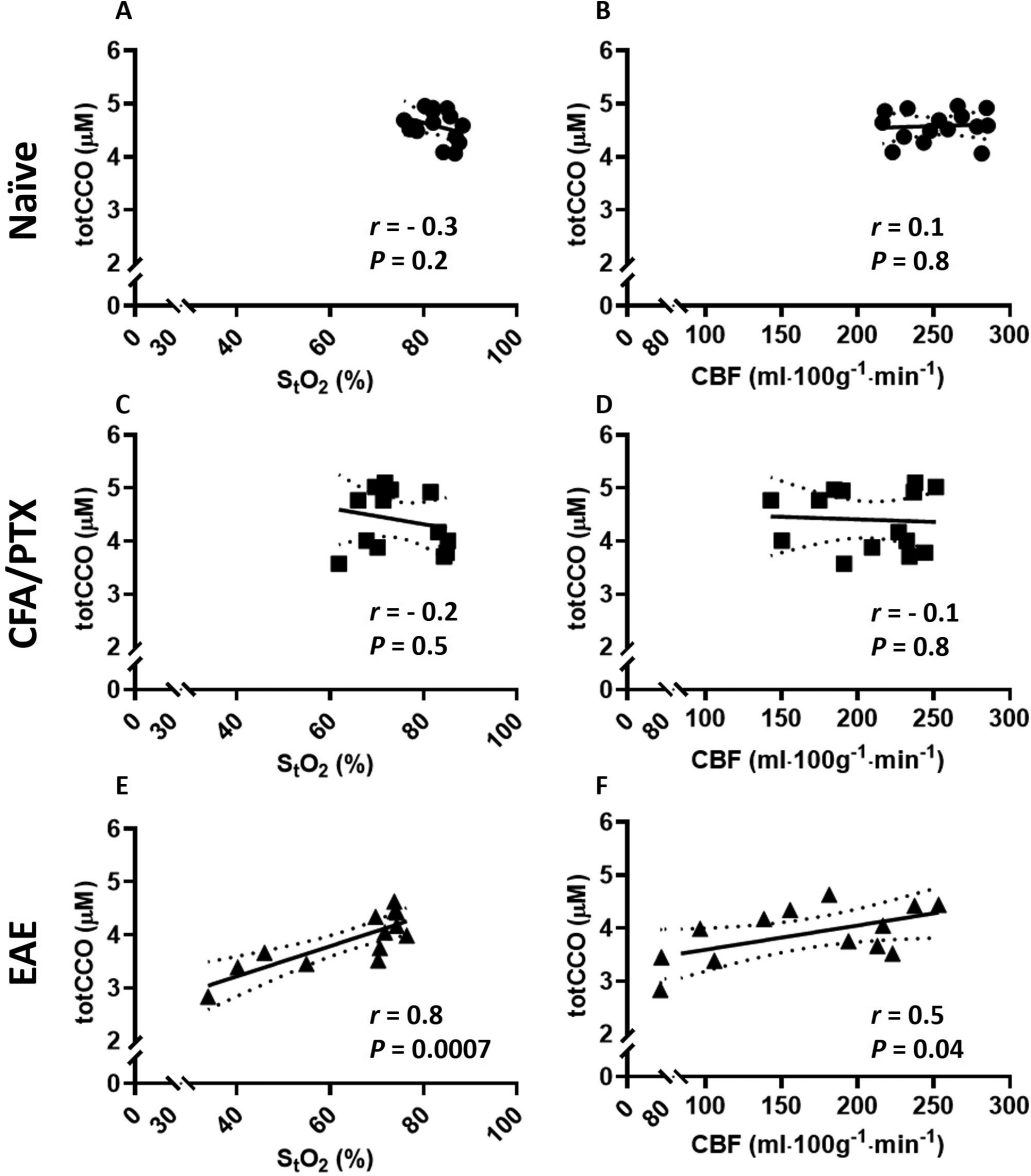
Correlation between totCCO, CBF, and S_t_O_2_ in naïve, CFA/PTX, and EAE mice. There was no significant correlation between totCCO, S_t_O_2_, and CBF in naïve (A, B) and CFA/PTX (C, D) mice. A significant positive correlation was found only in EAE mice (E, F) between totCCO, S_t_O_2_ (r = 0.8, *P* = 0.0007), and CBF (r = 0.5, *P* = 0.04). Each symbol represents a different animal (*n* = 15 naïve, 14 CFA/PTX, 13 EAE). Best fit line (solid line) and 95% confidence intervals (dotted lines) are plotted. Pearson correlation coefficients (r) and *p*-values (*P*) are displayed.

Figure 5A, 5C, and 5E shows the correlation between totCCO and S_t_O_2_ in naïve, CFA/PTX, and EAE mice. A significant positive correlation was found in EAE mice (*r* = 0.8, *P* = 0.0007) but not in naïve or CFA/PTX mice. EAE mice also showed a significant positive correlation (*r* = 0.5, *P* = 0.04) between totCCO and CBF (Fig. 5F). There was no significant correlation in naïve and CFA/PTX mice (Fig. 5B and 5D).

**Fig. 6.**
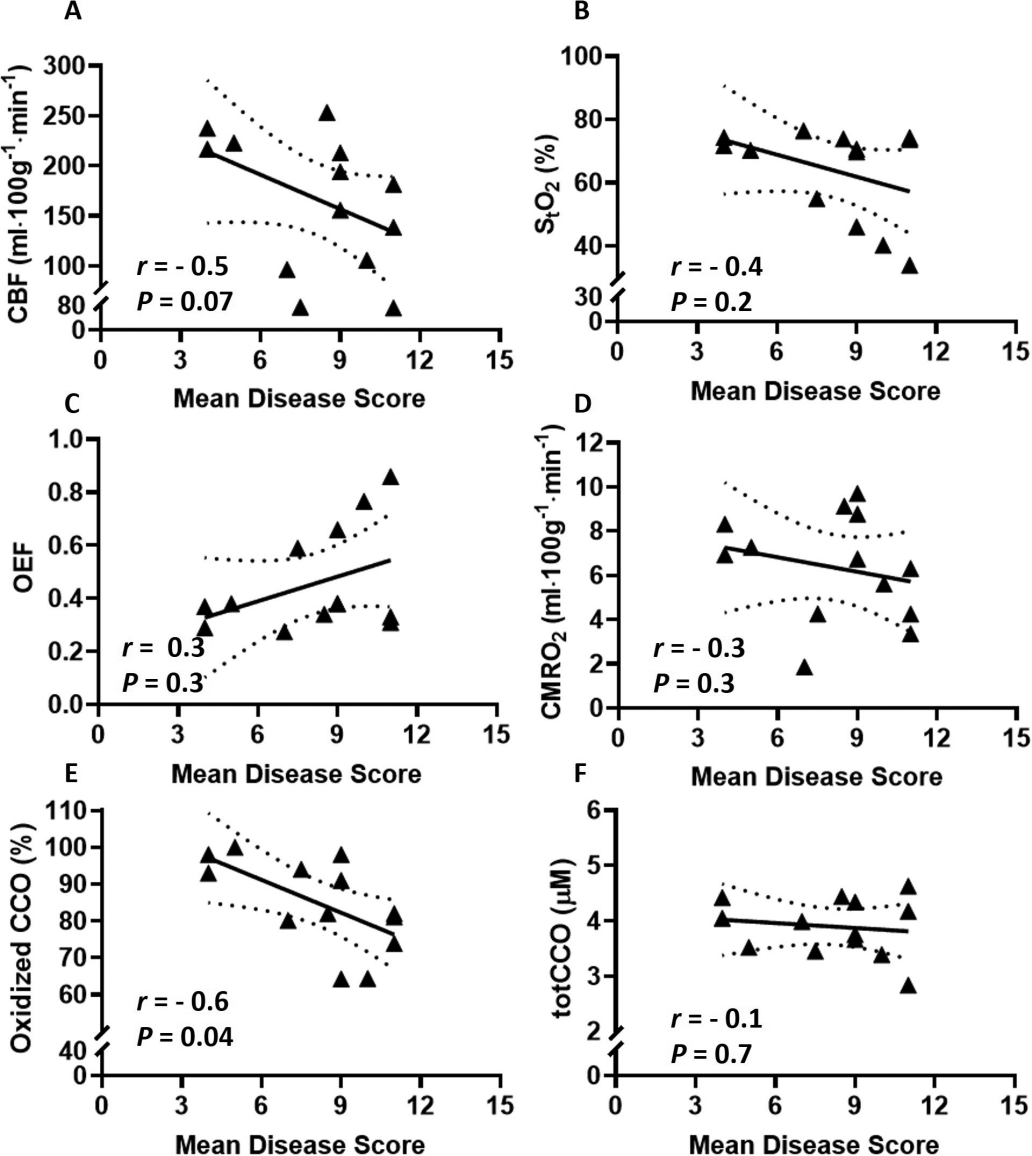
Correlation between the mean disease score of EAE mice and cortical oxidative metabolism metrics. Correlation between the mean disease score of EAE mice (*n* = 13) 15–17 days post-induction, and CBF (A), S_t_O_2_ (B), OEF (C), CMRO_2_ (D), oxidized CCO (E), and totCCO (F). Each symbol represents a different mouse. Best fit line (solid line) and 95% confidence intervals (dotted lines) are plotted. Pearson correlation coefficients (r) and *p*-values (*P*) are displayed.

To examine whether there is a direct association between the oxidative metabolism parameters measured in the cerebral cortex of EAE mice and physical disability, the mean disease score at peak disease was plotted against these metrics in Figure 6. There was a significant negative correlation only with the oxidation state of CCO (*r* = -0.6, *P* = 0.04). CBF showed a strong, but not statistically significant, negative correlation with the mean score (*r* = -0.5, *P* = 0.07).

**Fig. 7.**
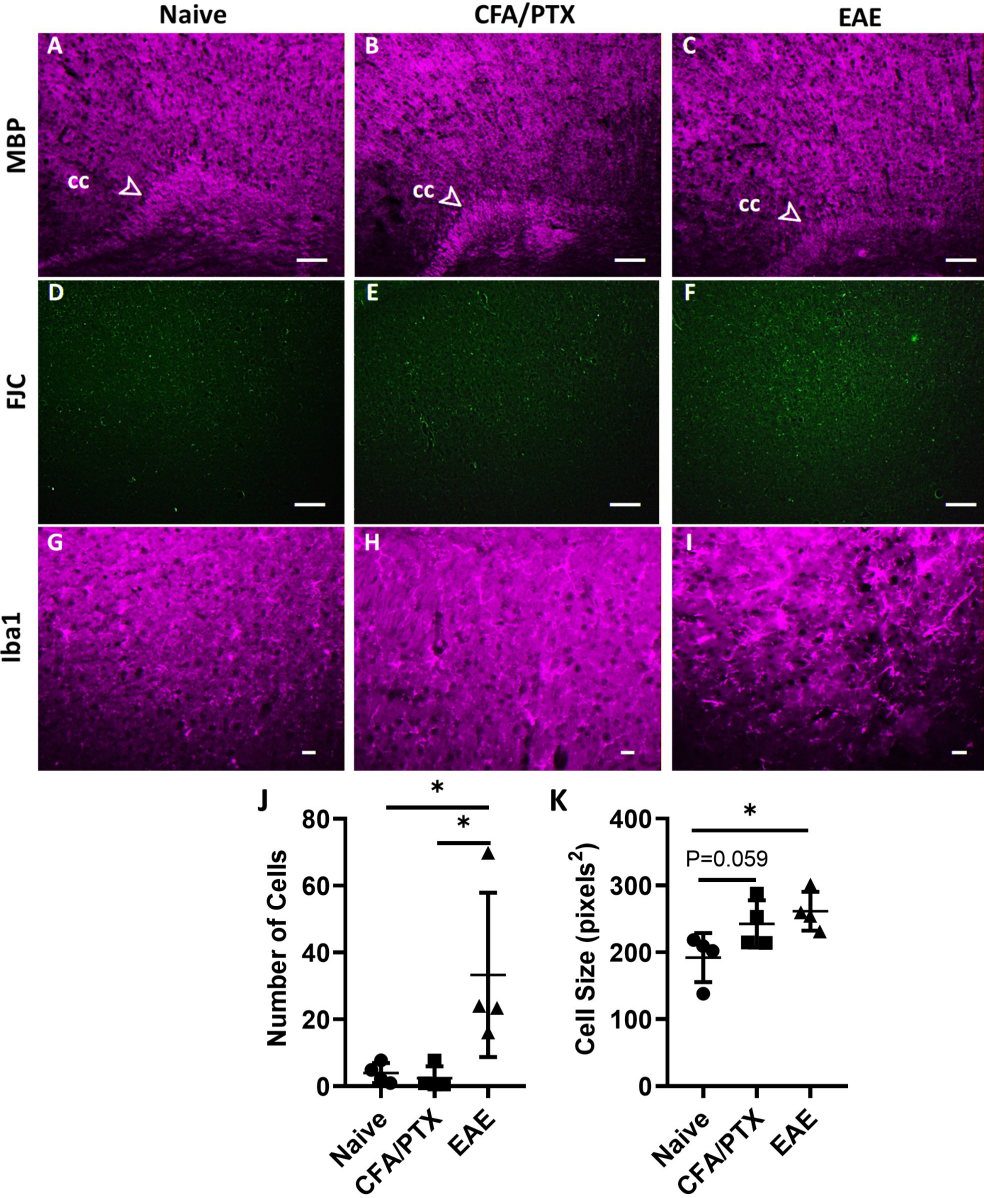
Cortical histology. Myelin basic protein (MBP) in the cerebral cortex and corpus callosum (cc) of naïve (A), CFA/PTX (B), and EAE (C) mice. cc (white arrow) serves as a reference region for evaluating myelin integrity. MBP staining shows well-defined myelin lines with no obvious differences between the groups, suggesting no significant myelin loss. A lack of Fluoro-Jade C (FJC) stain shows that there was no obvious neurodegeneration in naïve (D), CFA/PTX (E), and EAE (F) mice at peak disease. Increased microgliosis was seen by the elevated anti-ionized calcium-binding adapter molecule 1 (Iba1) staining in the cortex of EAE (I), compared with naïve (G) and CFA/PTX (H) mice. Magnification: 10X (MBP, FJC); 20X (Iba1). Scale bar: 50 µm. Quantification of the number of microglia cells (J) and cell size (K) stained with Iba1. Each symbol represents the mean of six sections imaged from the same mouse (*n* = 4 mice per group). Columns and error bars indicate mean and SD of each mouse group. Statistics were performed using RM one-way ANOVA, *P < 0.05.

The possibility of other cortical pathology such as demyelination, neurodegeneration, and inflammation accompanying the changes in oxidative metabolism in the cortex of EAE and CFA/PTX was determined with histology. Figure 7A–F shows no obvious demyelination or neurodegeneration present in the cerebral cortex of CFA/PTX and EAE at peak disease (15–17 days post-induction), as seen by the intact myelin basic protein (MBP) stain and the lack of Fluoro-Jade C (FJC) in the three mouse groups. We have previously shown that FJC selectively stains degenerating neurons in a nerve agent rat model (Lee et al., 2020). In contrast, as shown in Figure 7D–F, no significant FJC-positive cells were observed, indicating the absence of neuronal degeneration in the cerebral cortex of CFA/PTX and EAE mice at peak disease. EAE mice exhibited signs of microglia activation, with larger cell bodies seen in Iba1 staining (Fig. 7J), and higher numbers in the cortex compared with naïve (*P* = 0.02) and CFA/PTX (*P* = 0.01) mice. Moreover, the size of microglia in EAE was significantly higher than in naïve (*P* = 0.02) mice but comparable with those in CFA/PTX (*P* = 0.5) mice. Although the number of microglia was not higher in CFA/PTX mice, there was a trend toward a larger average microglial size compared with naïve mice (Fig. 7K; *P* = 0.059).

## 4 Discussion

**Fig. 8.**
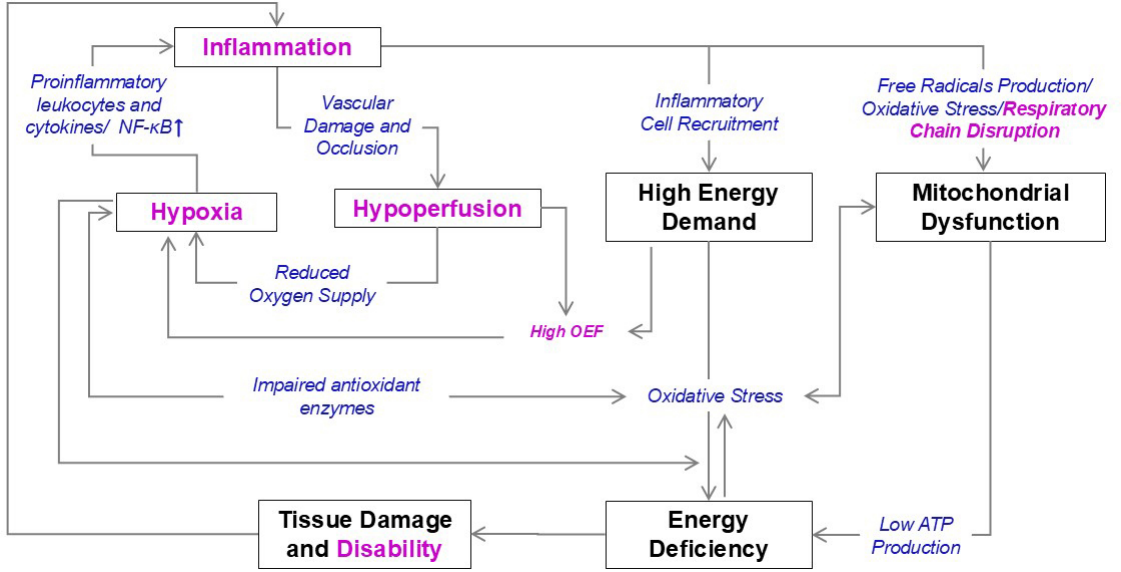
Potential linkages between inflammation, hypoxia, and tissue damage. Inflammation, hypoxia, hypoperfusion, oxidative damage to mitochondria, and altered energy production are all potential factors that could contribute to tissue degeneration and disability in MS. The scheme depicts major mechanisms associated with progression and atrophy (bold black) as well as physiological changes (blue) that link these mechanisms. Text in pink highlights what we found in the cortex of EAE at peak disease. Arrows indicate the direction of influence, with each arrow representing a contribution to the subsequent change. OEF—oxygen extraction fraction, ATP—adenosine triphosphate, NF-κB—nuclear factor-kappa B.

Multimodal NIRS–MRI revealed alterations in cortical oxidative metabolism of EAE and CFA/PTX mice. We found abnormal mitochondria in EAE mice at peak disease. In addition, other alterations, including hypoperfusion and hypoxia, were found in EAE and also in the CFA/PTX model of systemic inflammation. A complex interplay between inflammation, mitochondrial damage, and energy deficiency could contribute to disability and disease progression in EAE and MS. A schematic of these interactions is shown in Figure 8. It connects the changes we observed in EAE at peak disease with key mechanisms involved in MS progression and illustrates how these processes might collectively contribute to tissue damage. Below, we discuss each of the involved components in order.

### 4.1 Disease severity and cortical pathology in EAE at peak disease

EAE mice exhibited clear motor symptoms by day 12 and reached peak disease by days 15–17 post-induction. “Peak disease” refers to the time point of maximal disease symptom score, either reaching 10 or remaining stable for at least 2 days before day 17. There was no evidence of ongoing cell death or demyelination, as shown with the FJC and MBP staining, in EAE at this stage of pathology. This aligns with previous reports showing a delayed pattern of demyelination and neuronal loss that typically begins around 20 days and worsens by 39 days post-induction in cortical GM (Girolamo et al., 2011). In contrast, these changes happen earlier in the spinal cord, occurring as soon as 14–21 days after induction (Brown & Sawchenko, 2007; Girolamo et al., 2011). Longitudinal MRI data indicate that cortical atrophy develops progressively and is not present at early disease stages. Graham et al. reported that cerebral cortex volume declines gradually in EAE mice, with a significant ~5% reduction at ~80 days post-induction (mice were imaged at baseline and days 20, 40, and 80 post-induction). A substantial variability in atrophy rates across individual mice was also reported (MacKenzie-Graham et al., 2012). Hamilton et al. reported no cortical volume loss in EAE mice when imaged at peak disease (days 14–16 post-induction). However, they observed a significant ~3% decline in cerebral cortex volume in EAE mice with high disease scores at long-term disease (>66 days post-induction) (Hamilton et al., 2019).

Microgliosis was observed in the EAE cortex at peak disease, characterized by an elevated number of Iba1-stained microglia with an activated phenotype and larger cell bodies (Fig. 7I–K). Although CFA/PTX did not exhibit elevated microglia numbers, the stained microglia were comparable in size with those in EAE and larger than those in naïve mice. Alterations in morphology and increased size indicate heightened microglial activity, often associated with inflammatory responses (Vidal-Itriago et al., 2022). Alterations in morphology likely represent microglial activation in both CFA/PTX and EAE at peak disease (Vidal-Itriago et al., 2022). Increased numbers in EAE could indicate increased migration at peak disease. Mechanistic differences between EAE and CFA/PTX inflammatory responses may contribute to the observed differences in microglial cell size and number. CFA contains mycobacterial antigens that trigger strong innate immune activation and inflammation, inducing morphological changes (increased size) of microglia as a marker of activation, without a targeted autoimmune response and extensive infiltration of peripheral immune cells characteristic of EAE (Lazarević et al., 2024). The increase in microglial size induced by CFA/PTX occurs mainly through Toll-like receptor (TLR) pathways, leading to downstream activation of MAP kinase signaling and upregulation of proinflammatory cytokines (Biswas, 2023). This activation results in morphological changes of resident microglia without substantial proliferation or recruitment, as in EAE, explaining the discrepancy between cell size and cell number observed in microglial staining (Lazarević et al., 2024; Ronchi et al., 2016). In EAE, the presence of MOG-specific antigen, in addition to CFA and PTX, drives both microglial activation (increased size) and proliferation (increased cell number) through mechanisms involving IL-1β production and recruitment of encephalitogenic T cells and monocyte-derived cells (Ronchi et al., 2016).

### 4.2 Reduced cortical perfusion and oxygenation associated with inflammation

Cortical perfusion decreased in EAE at peak disease and CFA/PTX mice, resulting in reduced oxygen supply. Hypoperfusion has been shown in GM, WM, and normal-appearing WM (NAWM) of people with multiple sclerosis (pwMS) (Brooks et al., 1984; Law et al., 2004; Varga et al., 2009). Recent MRI studies have demonstrated hypoperfusion in brain regions of pwMS, without evidence of atrophy (Lapointe et al., 2018; Mascali et al., 2023; Vitorino et al., 2016).

Cortical microvasculature hypoxia was also detected, consistent with other studies. Cortical hypoxia was observed in 42% of pwMS, with hypoxia, defined as S_t_O_2_ levels being 2 SD below the average S_t_O_2_ of healthy controls (HC) (Yang & Dunn, 2015). In our study, S_t_O_2_ in 77% of EAE mice at peak disease and 64% of CFA/PTX mice were at least 2 SD below the average in naïve mice. Local hypoxia was previously reported in the cerebellum and cerebral cortex of EAE mice at peak disease using implanted pO_2_ probes (Johnson et al., 2016). Spinal cord hypoxia has been detected in EAE rats using pimonidazole labeling and confirmed *in vivo* with oxygen-sensitive probes (Davies et al., 2013; Desai et al., 2020). These findings are consistent with the low S_t_O_2_ level found in our EAE mice and confirm that hypoxia is a significant feature, not only in the spinal cord (Davies et al., 2013; Desai et al., 2020), but also in the cortex of the EAE inflammatory model.

Inflammation could be underlying hypoperfusion, particularly since hypoperfusion was also observed in CFA/PTX mice, which have systemic inflammation. CFA/PTX promotes inflammation by disrupting the blood–brain barrier, activating microglia, and facilitating the infiltration of effector T cells and macrophages (Munoz & Mackay, 1984; Zhao et al., 2008; Zou et al., 2022). Various mechanisms may contribute to the link between inflammation and hypoperfusion. Inflammation could damage cerebral blood vessels and lead to changes in blood flow regulation (D’haeseleer et al., 2011). Vascular abnormalities and endothelial dysfunction could alter the homeostatic properties of the endothelium and are secondary to inflammatory responses and oxidative stress (Cai & Harrison, 2000; D’haeseleer et al., 2011). Inflammation within the vessels could disrupt blood flow as leukocytes, platelets, and vascular adhesion molecules increase and directly occlude vessels (Chen et al., 2024; Wakefield et al., 1994). In MS, elevated nitric oxide (NO) levels resulting from inflammatory processes could also negatively impact CBF (Smith & Lassmann, 2002). Although NO is a vasodilator, its chronic overproduction can impair vascular function by desensitizing the endothelial and smooth muscle cells, thereby reducing the brain’s ability to regulate blood flow (Chiarelli et al., 2022; Marshall et al., 2014). This decreased vasodilatory capacity, resulting from repeated inflammatory cycles, can lead to low cerebrovascular reactivity (CVR), particularly in the GM that has a denser vascular network, linking inflammation to hypoperfusion (Marshall et al., 2014). Reduced CBF was also found in pwMS due to blood vessel constriction mediated by the production of vasospastic agents such as endothelin-1 (ET-1), which are stimulated by inflammatory processes (D’haeseleer et al., 2013).

Inflammation and hypoxia are strongly linked. Hypoxia is found in many inflammatory diseases (Eckle et al., 2013; Karhausen et al., 2004; Taccone et al., 2014). As EAE and CFA/PTX mice are inflammatory models, inflammation may be contributing to hypoxia, via the hypoxia–inflammation cycle we proposed (Yang & Dunn, 2019). We suggest hypoxia could be the result of either direct microvascular blockade by inflammatory cell infiltration or impaired vascular regulation due to inflammation. Other causes of tissue hypoxia could be the increased oxygen requirements of active glial cells. Some combination of hypoperfusion and normal or high oxygen demand could exceed the supply available, thus creating tissue hypoxia (Yang & Dunn, 2019).

### 4.3 Alterations in cerebral oxygen metabolism associated with inflammation

CMRO_2_ in EAE and CFA/PTX mice was comparable with that in naïve controls. As CBF decreased, reducing oxygen delivery, OEF increased to meet the brain’s metabolic demand. The common factor between EAE and CFA/PTX is the presence of inflammation. Even so, there was no significant increase in oxygen demand. It is possible that more severe inflammation could impact CMRO_2_, since CMRO_2_ in CFA/PTX and EAE mice was, although not reaching statistical significance (*P* = 0.5), higher by an average of 18% and 16%, respectively, than in naïve mice.

CMRO_2_ typically increases with increased brain activity and could be expected to increase due to increased metabolism caused by invading inflammatory cells. Some immune cells, however, alter their metabolic profile from mitochondrial respiration to glycolysis when activated, thus reducing CMRO_2_ (Xu et al., 2022). Thus, the impact on CMRO_2_ could vary depending on the nature and severity of inflammation, as different inflammatory responses could involve different immune cell populations and subpopulations.

Wide variations in CMRO_2_ and OEF were observed among pwMS, as measured with PET and TRUST-MRI (Brooks et al., 1984; Chandler et al., 2023; Ge et al., 2012; Sun et al., 1998; West et al., 2020). For instance, West et al. found that global CMRO_2_ was ~11% lower in pwMS than in HC; however, when grouped by CMRO_2_ quartiles, 25% of pwMS showed CMRO_2_ 12% higher than in HC. This metabolically elevated subgroup also showed lower fractional anisotropy (FA) in NAWM, a diffusion MRI measure that decreases when WM integrity is compromised, and worse fatigue and cognitive scores. The elevated CMRO_2_ was associated with greater WM damage and suggested ongoing demyelination and active autoimmune processes (West et al., 2020). Regional analyses of cortical and subcortical GM in pwMS have shown widespread reductions in CMRO_2_ without changes in OEF, compared with HC (Chandler et al., 2023). Decreases in CMRO_2_, ranging between 9 and 26%, were observed in both WM and cortical GM, with minimal or no change in OEF (Brooks et al., 1984; Ge et al., 2012; Sun et al., 1998). It has also been reported that different lesion phenotypes are associated with distinct metabolic signatures, with CMRO_2_ being 46.8% higher in active lesions than in inactive ones (Sivakolundu et al., 2019). A study focusing on lesions showed that OEF can vary between lesion types. It was higher in the rim of chronic active lesions and lower in paramagnetic rim lesions and hyperintense lesions. There were changes in OEF over time, reflecting lesion evolution (Xie et al., 2024). These variations indicate that oxygen levels may vary in GM over time and between pwMS, supporting the possibility of periodic hypoxia.

The variation in OEF among pwMS is consistent with the increased variation of S_t_O_2_ (cortical oxygenation) observed in EAE and CFA/PTX mice, with coefficients of variation (CV) of 23% in EAE, 11% in CFA/PTX vs. 5% in naïve controls (Fig. 3A). Similarly, implanted PO_2_ sensors in a previous study also demonstrated significant variability in EAE mice, with both hypoxic (ΔPO_2_ = -8.0 ± 4.6 mmHg) and hyperoxic (ΔPO_2_ = +0.8 ± 2.1 mmHg) days, reflecting variation both between animals and over time (Johnson et al., 2016). It is interesting to note that CBF (Fig. 2C) also showed increased variance in EAE and CFA/PTX (CV = 38% in EAE and 17% in CFA/PTX vs. CV = 10% in naïve controls). This provides supporting evidence that variations in CBF relate to the level of hypoxia.

Increased OEF in EAE and CFA/PTX, when perfusion is reduced, helps maintain oxygen supply to the mitochondria. This would result in low oxygen (hypoxia) in the microvasculature. Any further reduction in CBF could lead to reduced CMRO_2_ and exacerbate the disease, as OEF cannot increase any further.

### 4.4 Mitochondrial CCO alterations in EAE at peak disease

Interestingly, even though CMRO_2_ did not change, the concentration of CCO (totCCO) varied widely within EAE and CFA/PTX (CV = 13%) groups compared with naïve controls (CV = 6%), indicating that not all the mice exhibit the same changes at a given time. However, the average totCCO was significantly lower in EAE than in both CFA/PTX and naïve mice. Low totCCO could indicate abnormal mitochondria, fewer mitochondria per cell, or fewer living cells (G. Campbell & Mahad, 2018; Hatakeyama & Goto, 2017; D. Mahad et al., 2008). The absence of FJC staining in the cortex indicates no active degeneration. In the spinal cord, mitochondrial swelling was observed in morphologically normal axons 2–3 days after EAE symptom onset, before the appearance of axonal swellings or fragmentation that developed over subsequent hours to days (Nikić et al., 2011). Accordingly, we propose that low CCO content in the EAE cortex is more likely due to damaged mitochondria rather than cell death.

The significant positive correlation of totCCO with S_t_O_2_ and CBF in EAE only (Fig. 5), and not in CFA/PTX, shows that the alterations in oxygen metabolism in EAE could be attributed to mitochondrial abnormalities. The unique relationship between totCCO and S_t_O_2_ in EAE could also relate to the higher percentage of EAE mice experiencing hypoxia than CFA/PTX mice, and the physical disability occurring in EAE mice only.

Mitochondrial failure was associated with hypoxia and hypoperfusion in the spinal cord of EAE rats (Desai et al., 2020). Histological analysis of the spinal cord in EAE mice suggested a positive correlation between the exacerbation of mitochondrial dysfunction and the onset and worsening of physical disability (Sadeghian et al., 2016). Post-mortem studies in pwMS suggested that reactive oxygen species, produced by activated microglia and macrophages due to inflammation, induce mitochondrial dysfunction in both WM and GM lesions (Fischer et al., 2013). A deficiency in the respiratory chain components was identified in cortical NAWM of pwMS (G. R. Campbell et al., 2011). Neurons lacking mitochondrial DNA-encoded catalytic subunits of CCO were detected in cortical layer VI and subcortical WM of people with secondary progressive MS, irrespective of lesion presence (G. R. Campbell et al., 2011).

Both EAE and CFA/PTX mice displayed higher oxCCO than naïve mice. This provides evidence for mitochondrial oxidative stress and damage. Inflammation and immune activation, present in both models, have been shown to lead to impairment of enzymes in the Krebs cycle or upstream pathways, increased production of reactive oxygen species, disrupted electron transport chain activity, and loss of reducing agents, resulting in a shift toward the oxidized state of CCO (Pham et al., 2024; Qi et al., 2006; Steudler et al., 2022; Tai et al., 2023)*.* Depletion of critical upstream enzymes reduces NADH production, limiting electron flow through the respiratory chain and lowering CCO reduction capacity, thereby increasing its oxidation state (D. J. Mahad et al., 2009; Tai et al., 2023).

The oxidation state of CCO was negatively correlated with the mean disease score of EAE at peak disease. EAE with low disease scores had higher oxidation state, similar to CFA/PTX, while EAE with high disease scores (≥ 9) had low oxidation state. We propose that the lower oxidation state observed in more severely affected mice may reflect, in part, the inflammatory and oxidative injury to catalytic centers in CCO, which can shift the enzyme toward a more reduced state and impair its activity (Kowaltowski & Vercesi, 1999; Musatov & Robinson, 2012). It is also possible that EAE with higher disease scores experience more pronounced tissue hypoxia, causing reduced oxygen consumption, since a trend toward lower CBF, lower S_t_O_2_, and lower CMRO_2_ (although not statistically significant) was observed (Fig. 6). A more reduced state of CCO could compromise respiratory chain function and limit energy production, ultimately leading to mitochondrial failure. These observations are consistent with previous reports indicating that worsening mitochondrial function progresses in parallel with physical disability in EAE (Sadeghian et al., 2016).

Together with the low totCCO content in EAE, this negative correlation between oxCCO and disease score is consistent with progressive mitochondrial damage and energy failure. Since the EAE model shows more pronounced mitochondrial impairment than the CFA/PTX model (CFA/PTX had no reduction in totCCO), it is likely that some mitochondrial damage in EAE is due to autoimmune mechanisms (Atkinson et al., 2025; Licht‐Mayer et al., 2023; Srinivasan & Avadhani, 2012).

Our findings suggest that despite the altered oxidation state of CCO and the mitochondrial stress in CFA/PTX and EAE mice, CMRO_2_ was maintained at this disease stage through a combination of preserved mitochondrial respiration (Al Shamsi et al., 2021), compensatory metabolic responses (Packialakshmi & Zhou, 2018), and the high oxygen affinity of CCO during hypoxia and hypoperfusion (Harrison et al., 2015), until advanced disease stages lead to energy failure.

### 4.5 Inflammation, hypoxia, and mitochondrial dysfunction: interconnected factors in MS

Growing evidence shows that the causes of energy deficit in MS are multifactorial (Dutta et al., 2006; D. J. Mahad et al., 2009). Disability and progression of MS can be attributed to a complex interplay between immune activation, oxidative stress, and tissue damage (Fischer et al., 2013). Inflammation in MS and EAE mice triggers the release of soluble mediators, such as cytokines, which can impair mitochondrial function by disrupting the respiratory chain and reducing ATP production (D. H. Mahad et al., 2015). During acute inflammation, recruited inflammatory cells have a higher metabolic demand, which can increase oxidative stress and damage cellular components, contributing to tissue damage and disease progression. Inflammation can also lead to hypoxia, which can further exacerbate oxidative stress and tissue damage by impairing antioxidant enzyme function and promoting ROS production. Mitochondrial damage is implicated in most mechanisms linking inflammation and tissue loss. An energy deficit in GM could initiate a vicious cycle, leading to increased ROS production and accumulation of mtDNA damage (Druzhyna et al., 2008). Energy-deficient neurons could be more susceptible to inflammatory insults, as inflammation could exacerbate this energy deficit by increasing the metabolic demands of affected neurons (Trapp & Stys, 2009). An even more serious situation could arise in the event of hypoperfusion which leads to reduced energy substrate supply.

While there was no neuronal death, demyelination or atrophy observed at peak disease, inflammation, and physiological changes, including decreased perfusion, lower S_t_O_2_, higher OEF, and altered mitochondrial function, occurred in the cerebral cortex. In summary, inflammation, hypoxia, oxidative damage to mitochondria, and altered energy production are all potential factors that can contribute to tissue degeneration and disability in MS (Fig. 8).

### 4.5 Study limitations

Although we observe significant changes in perfusion and other oxygen metabolism metrics in the cortical GM of EAE, we lack the data to identify the underlying mechanisms. As such, we are limited to suggesting mechanisms that may explain the link between these changes and inflammation. Additionally, in our histological analysis, Iba1 staining and quantification show significant microglia activation in EAE, suggesting inflammation and immune response involvement. However, despite the absence of significant MBP loss and FJC staining, this does not rule out the possibility of subtle demyelination or neurodegeneration that could be occurring at a level undetectable with these stains. Furthermore, the relatively low number of mice used for histology in this study limits the statistical power of our findings. These limitations should be addressed in future work to better understand the complex link between hypoperfusion, hypoxia, mitochondrial damage, and inflammation, and their contribution to tissue damage and disease progression.

## 5 Conclusions

This study is the first to investigate perfusion and hypoxia concurrently with *in vivo* mitochondrial function and CMRO_2_ in the cerebral cortex of EAE mice. Using a novel NIRS–MRI multimodality system, we detected significant metabolic alterations. We suggest that reduced perfusion and oxygen supply were caused by inflammation. Reduced oxygen supply with no change in CMRO_2_ could lead to severe hypoxia. There also appears to be mitochondrial dysfunction in EAE mice. While inflammation might be the main contributor to oxidative metabolism changes in CFA/PTX, inflammation and mitochondrial damage play a significant role in EAE mice at peak disease. Damaged mitochondria, long-term hypoxia, and inefficient energy production are likely to exacerbate GM pathology in MS. The ability to non-invasively measure all these parameters simultaneously in EAE allows for better understanding of the physiological changes contributing to tissue degeneration and disease progression. These findings help understand the pathogenesis of MS and other CNS diseases and could help develop effective therapeutic strategies. For instance, approaches aimed at protecting and maintaining mitochondrial health would have potential benefits. Additionally, reducing hypoxia and enhancing CBF could be done through a range of options including oxygen therapy and vasodilators (Soroush & Dunn, 2025).

## Data Availability

Datasets generated during the current study are available in PRISM Dataverse: University of Calgary’s Data Repository, at https://doi.org/10.5683/SP3/NXTMBA. More data may be available upon reasonable request from the corresponding author.

## References

[IMAG.a.1192-b1] Acharjee, S., Nayani, N., Tsutsui, M., Hill, M. N., Ousman, S. S., & Pittman, Q. J. (2013). Altered cognitive-emotional behavior in early experimental autoimmune encephalitis–cytokine and hormonal correlates. Brain, Behavior, and Immunity, 33, 164–172. 10.1016/j.bbi.2013.07.003 23886782

[IMAG.a.1192-b2] Al Shamsi, M., Shahin, A., Kamyan, D., Alnaqbi, A., Shaban, S., & Souid, A.-K. (2021). Conserved spinal cord bioenergetics in experimental autoimmune encephalomyelitis in C57BL6 mice, measured using phosphorescence oxygen analyzer. Heliyon, 7(10). 10.1016/j.heliyon.2021.e08111 PMC8511844 34693048

[IMAG.a.1192-b3] Alizadeh, A., Dyck, S. M., & Karimi-Abdolrezaee, S. (2015). Myelin damage and repair in pathologic CNS: Challenges and prospects. Frontiers in Molecular Neuroscience, 8, 35. 10.3389/fnmol.2015.00035 26283909 PMC4515562

[IMAG.a.1192-b4] Ames III, A. (2000). CNS energy metabolism as related to function. Brain Research Reviews, 34(1–2), 42–68. 10.1016/s0165-0173(00)00038-2 11086186

[IMAG.a.1192-b5] Atkinson, K. C., Desfor, S., Feri, M., Sekyi, M. T., Osunde, M., Sriram, S., Noori, S., Rincón, W., Bello, B., & Tiwari-Woodruff, S. K. (2025). Decreased mitochondrial activity in the demyelinating cerebellum of progressive multiple sclerosis and chronic EAE contributes to Purkinje cell loss. Proceedings of the National Academy of Sciences, 122(25), e2421806122. 10.1073/pnas.2421806122 PMC12207518 40523174

[IMAG.a.1192-b6] Baecher-Allan, C., Kaskow, B. J., & Weiner, H. L. (2018). Multiple sclerosis: Mechanisms and immunotherapy. Neuron, 97(4), 742–768. 10.1016/j.neuron.2018.01.021 29470968

[IMAG.a.1192-b7] Bashkatov, A. N., Genina, E. A., Sinichkin, Y. P., & Tuchin, V. V. (2002). Influence of glycerol on the transport of light in the skin. Paper presented at the Functional monitoring and drug-tissue interaction. 10.1117/12.469442

[IMAG.a.1192-b8] Biswas, K. (2023). Microglia mediated neuroinflammation in neurodegenerative diseases: A review on the cell signaling pathways involved in microglial activation. Journal of Neuroimmunology, 383, 578180. 10.1016/j.jneuroim.2023.578180 37672840

[IMAG.a.1192-b9] Brooks, D., Leenders, K., Head, G., Marshall, J., Legg, N., & Jones, T. (1984). Studies on regional cerebral oxygen utilisation and cognitive function in multiple sclerosis. Journal of Neurology, Neurosurgery & Psychiatry, 47(11), 1182–1191. 10.1136/jnnp.47.11.1182 6334132 PMC1028084

[IMAG.a.1192-b10] Brown, D. A., & Sawchenko, P. E. (2007). Time course and distribution of inflammatory and neurodegenerative events suggest structural bases for the pathogenesis of experimental autoimmune encephalomyelitis. Journal of Comparative Neurology, 502(2), 236–260. 10.1002/cne.21307 17348011

[IMAG.a.1192-b11] Cai, H., & Harrison, D. G. (2000). Endothelial dysfunction in cardiovascular diseases: The role of oxidant stress. Circulation Research, 87(10), 840–844. 10.1161/01.res.87.10.840 11073878

[IMAG.a.1192-b12] Campbell, G., & Mahad, D. J. (2018). Mitochondrial dysfunction and axon degeneration in progressive multiple sclerosis. FEBS Letters, 592(7), 1113–1121. 10.1002/1873-3468.13013 29453889

[IMAG.a.1192-b13] Campbell, G. R., Ziabreva, I., Reeve, A. K., Krishnan, K. J., Reynolds, R., Howell, O., Lassmann, H., Turnbull, D. M., & Mahad, D. J. (2011). Mitochondrial DNA deletions and neurodegeneration in multiple sclerosis. Annals of Neurology, 69(3), 481–492. 10.1002/ana.22109 21446022 PMC3580047

[IMAG.a.1192-b14] Chanaday, N., & Roth, G. (2016). Microglia and astrocyte activation in the frontal cortex of rats with experimental autoimmune encephalomyelitis. Neuroscience, 314, 160–169. 10.1016/j.neuroscience.2015.11.060 26679600

[IMAG.a.1192-b15] Chandler, H. L., Stickland, R. C., Patitucci, E., Germuska, M., Chiarelli, A. M., Foster, C., Bhome-Dhaliwal, S., Lancaster, T. M., Saxena, N., Khot, S., Tomassini, V., & Wise, R. G. (2023). Reduced brain oxygen metabolism in patients with multiple sclerosis: Evidence from dual-calibrated functional MRI. Journal of Cerebral Blood Flow & Metabolism, 43(1), 115–128. 10.1177/0271678x221121849 36071645 PMC9875355

[IMAG.a.1192-b16] Chen, T., Dai, Y., Hu, C., Lin, Z., Wang, S., Yang, J., Zeng, L., Li, S., & Li, W. (2024). Cellular and molecular mechanisms of the blood–brain barrier dysfunction in neurodegenerative diseases. Fluids and Barriers of the CNS, 21(1), 60. 10.1186/s12987-024-00557-1 39030617 PMC11264766

[IMAG.a.1192-b17] Chiarelli, A. M., Villani, A., Mascali, D., Petsas, N., Biondetti, E., Caporale, A., Digiovanni, A., Grasso, E., Paola Ajdinaj, M., d’Apolito, M., Rispoli, M., Sensi, S., Murphy, K., Pozzilli, C., Wise, R., & Tomassini, V. (2022). Cerebrovascular reactivity in multiple sclerosis is restored with reduced inflammation during immunomodulation. Scientific Reports, 12(1), 15453. 10.1038/s41598-022-19113-8 36104366 PMC9474533

[IMAG.a.1192-b18] Cooper, C. E., Delpy, D. T., & Nemoto, E. M. (1998). The relationship of oxygen delivery to absolute haemoglobin oxygenation and mitochondrial cytochrome oxidase redox state in the adult brain: A near-infrared spectroscopy study. The Biochemical Journal, 332 (Pt 3)(Pt 3), 627–632. 10.1042/bj3320627 9620863 PMC1219521

[IMAG.a.1192-b19] D’haeseleer, M., Beelen, R., Fierens, Y., Cambron, M., Vanbinst, A.-M., Verborgh, C., Demey J., & De Keyser, J. (2013). Cerebral hypoperfusion in multiple sclerosis is reversible and mediated by endothelin-1. Proceedings of the National Academy of Sciences, 110(14), 5654–5658. 10.1073/pnas.1222560110 PMC3619305 23509249

[IMAG.a.1192-b20] D’haeseleer, M., Cambron, M., Vanopdenbosch, L., & De Keyser, J. (2011). Vascular aspects of multiple sclerosis. The Lancet Neurology, 10(7), 657–666. 10.1016/s1474-4422(11)70105-3 21683931

[IMAG.a.1192-b21] D’haeseleer, M., Hostenbach, S., Peeters, I., Sankari, S. E., Nagels, G., De Keyser, J., & D’hooghe, M. B. (2015). Cerebral hypoperfusion: A new pathophysiologic concept in multiple sclerosis? Journal of Cerebral Blood Flow & Metabolism, 35(9), 1406–1410. 10.1038/jcbfm.2015.131 26104292 PMC4640326

[IMAG.a.1192-b22] Davies, A. L., Desai, R. A., Bloomfield, P. S., McIntosh, P. R., Chapple, K. J., Linington, C., Fairless, R., Diem, R., Kasti, M., Murphy, M. P., & Smith, K. J. (2013). Neurological deficits caused by tissue hypoxia in neuroinflammatory disease. Annals of Neurology, 74(6), 815–825. 10.1002/ana.24006 24038279

[IMAG.a.1192-b23] Desai, R. A., Davies, A. L., Del Rossi, N., Tachrount, M., Dyson, A., Gustavson, B., Kaynezhad, P., Mackenzie, L., van der Putten, M. A., McElroy, D., Schiza, D., Linington, C., Singer, M., Harvey, A. R., Tachtsidis, I., Golay, X., & Smith, K. J. (2020). Nimodipine reduces dysfunction and demyelination in models of multiple sclerosis. Annals of Neurology, 88(1), 123–136. 10.1002/ana.25749 32293054 PMC7737229

[IMAG.a.1192-b24] Druzhyna, N. M., Wilson, G. L., & LeDoux, S. P. (2008). Mitochondrial DNA repair in aging and disease. Mechanisms of Ageing and Development, 129(7–8), 383–390. 10.1016/j.mad.2008.03.002 18417187 PMC2666190

[IMAG.a.1192-b25] Dutta, R., McDonough, J., Yin, X., Peterson, J., Chang, A., Torres, T., Gudz, T., Macklin, W. B., Lewis, D. A., Fox, R. J., Rudick, R., Mirnics, K., & Trapp, B. D. (2006). Mitochondrial dysfunction as a cause of axonal degeneration in multiple sclerosis patients. Annals of Neurology, 59(3), 478–489. 10.1002/ana.20736 16392116

[IMAG.a.1192-b26] Eckle, T., Brodsky, K., Bonney, M., Packard, T., Han, J., Borchers, C. H., Mariani, T. J., Kominsky, D. J., Mittelbronn, M., & Eltzschig, H. K. (2013). HIF1A reduces acute lung injury by optimizing carbohydrate metabolism in the alveolar epithelium. PLoS Biology, 11(9), e1001665. 10.1371/journal.pbio.1001665 24086109 PMC3782424

[IMAG.a.1192-b27] Feigin, V. L., Nichols, E., Alam, T., Bannick, M. S., Beghi, E., Blake, N., Culpepper, W. J., Ray Dorsey, E., Elbaz, A., Ellenbogen, R. G., Fisher, J. L., Fitzmaurice, C., Giussani, G., Glennie, L., James, S. L., Johnson, C. O., Kassebaum, N. J., Logroscino, G., Marin, B.,… Vos, T. (2019). Global, regional, and national burden of neurological disorders, 1990–2016: A systematic analysis for the Global Burden of Disease Study 2016. The Lancet Neurology, 18(5), 459–480. 10.3410/f.735346535.793561284 30879893 PMC6459001

[IMAG.a.1192-b28] Fischer, M. T., Wimmer, I., Höftberger, R., Gerlach, S., Haider, L., Zrzavy, T., Hametner, S., Mahad, D., Binder, C. J., Krumbholz, M., Bauer, J., Bradl, M., & Lassmann, H. (2013). Disease-specific molecular events in cortical multiple sclerosis lesions. Brain, 136(6), 1799–1815. 10.1093/brain/awt110 23687122 PMC3673462

[IMAG.a.1192-b29] Ge, Y., Zhang, Z., Lu, H., Tang, L., Jaggi, H., Herbert, J., Babb, J. S., Rusinek, H., & Grossman, R. I. (2012). Characterizing brain oxygen metabolism in patients with multiple sclerosis with T2-relaxation-under-spin-tagging MRI. Journal of Cerebral Blood Flow & Metabolism, 32(3), 403–412. 10.1038/jcbfm.2011.191 22252237 PMC3293125

[IMAG.a.1192-b30] Gerevich, Z., Kovács, R., Liotta, A., Hasam-Henderson, L. A., Weh, L., Wallach, I., & Berndt, N. (2023). Metabolic implications of axonal demyelination and its consequences for synchronized network activity: An in silico and in vitro study. Journal of Cerebral Blood Flow & Metabolism, 43(9), 1571–1587. 10.1177/0271678x231170746 37125487 PMC10414014

[IMAG.a.1192-b31] Geurts, J. J., & Barkhof, F. (2008). Grey matter pathology in multiple sclerosis. The Lancet Neurology, 7(9), 841–851. 10.1016/s1474-4422(08)70191-1 18703006

[IMAG.a.1192-b32] Girolamo, F., Ferrara, G., Strippoli, M., Rizzi, M., Errede, M., Trojano, M., Perris, R., Roncali, L., Svelto, M., Mennini, T., & Virgintino, D. (2011). Cerebral cortex demyelination and oligodendrocyte precursor response to experimental autoimmune encephalomyelitis. Neurobiology of Disease, 43(3), 678–689. 10.1016/j.nbd.2011.05.021 21679768

[IMAG.a.1192-b33] Haider, L., Simeonidou, C., Steinberger, G., Hametner, S., Grigoriadis, N., Deretzi, G., Kovacs, G. G., Kutzelnigg, A., Lassmann, H., & Frischer, J. M. (2014). Multiple sclerosis deep grey matter: The relation between demyelination, neurodegeneration, inflammation and iron. Journal of Neurology, Neurosurgery & Psychiatry, 85(12), 1386–1395. 10.1136/jnnp-2014-307712 24899728 PMC4251183

[IMAG.a.1192-b34] Hamilton, A. M., Forkert, N. D., Yang, R., Wu, Y., Rogers, J. A., Yong, V. W., & Dunn, J. F. (2019). Central nervous system targeted autoimmunity causes regional atrophy: A 9.4 T MRI study of the EAE mouse model of Multiple Sclerosis. Scientific Reports, 9(1), 1–13. 10.1038/s41598-019-44682-6 31186441 PMC6560061

[IMAG.a.1192-b35] Harrison, D. K., Fasching, M., Fontana-Ayoub, M., & Gnaiger, E. (2015). Cytochrome redox states and respiratory control in mouse and beef heart mitochondria at steady-state levels of hypoxia. Journal of Applied Physiology, 119(10), 1210–1218. 10.1152/japplphysiol.00146.2015 26251509

[IMAG.a.1192-b36] Hashem, M., Shafqat, Q., Wu, Y., Rho, J. M., & Dunn, J. F. (2022). Abnormal oxidative metabolism in the cuprizone mouse model of demyelination: An in vivo NIRS-MRI study. NeuroImage, 250, 118935. 10.1016/j.neuroimage.2022.118935 35091079

[IMAG.a.1192-b37] Hashem, M., Wu, Y., & Dunn, J. F. (2023). The relationship between cytochrome c oxidase, CBF and CMRO2 in mouse cortex: A NIRS-MRI study. Journal of Cerebral Blood Flow & Metabolism, 43(8), 1351–1364. 10.1177/0271678x231165842 36950950 PMC10369142

[IMAG.a.1192-b38] Hashem, M., Wu, Y., & Dunn, J. F. (2021). Quantification of cytochrome c oxidase and tissue oxygenation using CW-NIRS in a mouse cerebral cortex. Biomedical Optics Express, 12(12), 7632–7656. 10.1364/boe.435532 35003857 PMC8713667

[IMAG.a.1192-b39] Hashem, M., Zhang, Q., Wu, Y., Johnson, T. W., & Dunn, J. F. (2020). Using a multimodal near-infrared spectroscopy and MRI to quantify gray matter metabolic rate for oxygen: A hypothermia validation study. Neuroimage, 206, 116315. 10.1016/j.neuroimage.2019.116315 31669409 PMC6983321

[IMAG.a.1192-b40] Hatakeyama, H., & Goto, Y.-I. (2017). Respiratory chain complex disorganization impairs mitochondrial and cellular integrity: Phenotypic variation in cytochrome c oxidase deficiency. The American Journal of Pathology, 187(1), 110–121. 10.1016/j.ajpath.2016.09.003 27855277

[IMAG.a.1192-b41] Jakimovski, D., Bittner, S., Zivadinov, R., Morrow, S. A., Benedict, R. H., Zipp, F., & Weinstock-Guttman, B. (2024). Multiple sclerosis. Lancet, 403(10422), 183–202. 10.1016/s0140-6736(23)01473-3 37949093

[IMAG.a.1192-b42] Johnson, T. W. (2017). Measurement of brain oxygenation and metabolism in a mouse model of multiple sclerosis. A Thesis Submitted to the Faculty of Graduate Studies in Partial Fulfillment of the Requirements for the Degree of Doctor of Philosophy. 10.26226/morressier.59a3eda2d462b8028d894843

[IMAG.a.1192-b43] Johnson, T. W., Wu, Y., Nathoo, N., Rogers, J. A., Wee Yong, V., & Dunn, J. F. (2016). Gray matter hypoxia in the brain of the experimental autoimmune encephalomyelitis model of multiple sclerosis. PLoS One, 11(12), e0167196. 10.1371/journal.pone.0167196 27907119 PMC5131950

[IMAG.a.1192-b44] Karhausen, J., Furuta, G. T., Tomaszewski, J. E., Johnson, R. S., Colgan, S. P., & Haase, V. H. (2004). Epithelial hypoxia-inducible factor-1 is protective in murine experimental colitis. The Journal of Clinical Investigation, 114(8), 1098–1106. 10.1172/jci200421086 15489957 PMC522241

[IMAG.a.1192-b45] Kety, S. S., & Schmidt, C. F. (1948). The nitrous oxide method for the quantitative determination of cerebral blood flow in man: Theory, procedure and normal values. The Journal of Clinical Investigation, 27(4), 476–483. 10.1172/jci101994 16695568 PMC439518

[IMAG.a.1192-b46] Klaver, R., De Vries, H. E., Schenk, G. J., & Geurts, J. J. (2013). Grey matter damage in multiple sclerosis: A pathology perspective. Prion, 7(1), 66–75. 10.4161/pri.23499 23324595 PMC3609053

[IMAG.a.1192-b47] Kowaltowski, A. J., & Vercesi, A. E. (1999). Mitochondrial damage induced by conditions of oxidative stress. Free Radical Biology and Medicine, 26(3–4), 463–471. 10.1016/s0891-5849(98)00216-0 9895239

[IMAG.a.1192-b48] Lapointe, E., Li, D., Traboulsee, A., & Rauscher, A. (2018). What have we learned from perfusion MRI in multiple sclerosis? American Journal of Neuroradiology, 39(6), 994–1000. 10.3174/ajnr.a5504 29301779 PMC7410640

[IMAG.a.1192-b49] Law, M., Saindane, A. M., Ge, Y., Babb, J. S., Johnson, G., Mannon, L. J., Herbert, J., & Grossman, R. I. (2004). Microvascular abnormality in relapsing-remitting multiple sclerosis: Perfusion MR imaging findings in normal-appearing white matter. Radiology, 231(3), 645–652. 10.1148/radiol.2313030996 15163806

[IMAG.a.1192-b50] Lazarević, M., Stanisavljević, S., Nikolovski, N., Dimitrijević, M., & Miljković, Đ. (2024). Complete Freund’s adjuvant as a confounding factor in multiple sclerosis research. Frontiers in Immunology, 15, 1353865. 10.3389/fimmu.2024.1353865 38426111 PMC10902151

[IMAG.a.1192-b51] Lee, K., Bohnert, S., Bouchard, M., Vair, C., Farrell, J. S., Teskey, G. C., Mikler, J., & Dunn, J. F. (2020). Quantitative T2 MRI is predictive of neurodegeneration following organophosphate exposure in a rat model. Scientific Reports, 10(1), 13007. 10.1038/s41598-020-69991-z 32747689 PMC7400670

[IMAG.a.1192-b52] Licht‐Mayer, S., Campbell, G. R., Mehta, A. R., McGill, K., Symonds, A., Al‐Azki, S., Pryce, G., Zandee, S., Zhao, C., Kipp, M., Smith, K. J., Baker, D., Altmann, D., Anderton, S. M., Kap, Y. S., Laman, J. D., 't Hart, B. A., Rodriguez, M., Franklin, R. J. M., … Mahad, D. J. (2023). Axonal response of mitochondria to demyelination and complex IV activity within demyelinated axons in experimental models of multiple sclerosis. Neuropathology and Applied Neurobiology, 49(1), e12851. 10.1111/nan.12851 36181265 PMC10092519

[IMAG.a.1192-b53] MacKenzie-Graham, A., Rinek, G. A., Avedisian, A., Gold, S. M., Frew, A. J., Aguilar, C., Lin, D. R., Umeda, E., Voskuhl, R. R., & Alger, J. R. (2012). Cortical atrophy in experimental autoimmune encephalomyelitis: In vivo imaging. NeuroImage, 60(1), 95–104. 10.1016/j.neuroimage.2011.11.099 22182769 PMC3293104

[IMAG.a.1192-b54] Mahad, D., Ziabreva, I., Lassmann, H., & Turnbull, D. (2008). Mitochondrial defects in acute multiple sclerosis lesions. Brain, 131(7), 1722–1735. 10.1093/brain/awn105 18515320 PMC2442422

[IMAG.a.1192-b55] Mahad, D. H., Trapp, B. D., & Lassmann, H. (2015). Pathological mechanisms in progressive multiple sclerosis. The Lancet Neurology, 14(2), 183–193. 10.1016/s1474-4422(14)70256-x 25772897

[IMAG.a.1192-b56] Mahad, D. J., Ziabreva, I., Campbell, G., Lax, N., White, K., Hanson, P. S., Lassmann, H., & Turnbull, D. M. (2009). Mitochondrial changes within axons in multiple sclerosis. Brain, 132(5), 1161–1174. 10.1093/brain/awp046 19293237 PMC3605917

[IMAG.a.1192-b57] Marshall, O., Lu, H., Brisset, J.-C., Xu, F., Liu, P., Herbert, J., Grossman, R. I., & Ge, Y. (2014). Impaired cerebrovascular reactivity in multiple sclerosis. JAMA Neurology, 71(10), 1275–1281. 10.1001/jamaneurol.2014.1668 25133874 PMC4376108

[IMAG.a.1192-b58] Mascali, D., Villani, A., Chiarelli, A. M., Biondetti, E., Lipp, I., Digiovanni, A., Pozzilli, V., Caporale, A. S., Rispoli, M. G., Ajdinaj, P., D’Apolito, M., Grasso, E., Sensi, S. L., Murphy, K., Tomassini, V., & Wise, R. G. (2023). Pathophysiology of multiple sclerosis damage and repair: Linking cerebral hypoperfusion to the development of irreversible tissue loss in multiple sclerosis using magnetic resonance imaging. European Journal of Neurology, 30(8), 2348–2356. 10.1111/ene.15827 37154298 PMC7615142

[IMAG.a.1192-b59] Matcher, S. J., & Cooper, C. E. (1994). Absolute quantification of deoxyhaemoglobin concentration in tissue near infrared spectroscopy. Physics in Medicine & Biology, 39(8), 1295–1312. 10.1088/0031-9155/39/8/008 15551568

[IMAG.a.1192-b60] Matcher, S. J., Cope, M., & Delpy, D. T. (1994). Use of the water absorption spectrum to quantify tissue chromophore concentration changes in near-infrared spectroscopy. Physics in Medicine & Biology, 39(1), 177–196. 10.1088/0031-9155/39/1/011 7651995

[IMAG.a.1192-b61] McCombe, P. A., & Greer, J. M. (2022). Effects of biological sex and pregnancy in experimental autoimmune encephalomyelitis: It’s complicated. Frontiers in Immunology, 13, 1059833. 10.3389/fimmu.2022.1059833 36518769 PMC9742606

[IMAG.a.1192-b62] Munoz, J., & Mackay, I. (1984). Adoptive transfer of experimental allergic encephalomyelitis in mice with the aid of pertussigen from Bordetella pertussis. Cellular Immunology, 86(2), 541–545. 10.1016/0008-8749(84)90410-6 6329525

[IMAG.a.1192-b63] Musatov, A., & Robinson, N. C. (2012). Susceptibility of mitochondrial electron-transport complexes to oxidative damage. Focus on cytochrome c oxidase. Free Radical Research, 46(11), 1313–1326. 10.3109/10715762.2012.717273 22856385

[IMAG.a.1192-b64] Nikić, I., Merkler, D., Sorbara, C., Brinkoetter, M., Kreutzfeldt, M., Bareyre, F. M., Brück, W., Bishop, D., Misgeld, T., & Kerschensteiner, M. (2011). A reversible form of axon damage in experimental autoimmune encephalomyelitis and multiple sclerosis. Nature Medicine, 17(4), 495–499. 10.1038/nm.2324 21441916

[IMAG.a.1192-b65] Packialakshmi, B., & Zhou, X. (2018). Experimental autoimmune encephalomyelitis (EAE) up-regulates the mitochondrial activity and manganese superoxide dismutase (MnSOD) in the mouse renal cortex. PLoS One, 13(4), e0196277. 10.1371/journal.pone.0196277 29689072 PMC5916489

[IMAG.a.1192-b66] Pham, L., Arroum, T., Wan, J., Pavelich, L., Bell, J., Morse, P. T., Lee, I., Grossman, L. I., Sanderson, T. H., Malek, M. H., & Hüttemann, M. (2024). Regulation of mitochondrial oxidative phosphorylation through tight control of cytochrome c oxidase in health and disease–Implications for ischemia/reperfusion injury, inflammatory diseases, diabetes, and cancer. Redox Biology, 78, 103426. 10.1016/j.redox.2024.103426 39566165 PMC11617887

[IMAG.a.1192-b67] Qi, X., Lewin, A. S., Sun, L., Hauswirth, W. W., & Guy, J. (2006). Mitochondrial protein nitration primes neurodegeneration in experimental autoimmune encephalomyelitis. Journal of Biological Chemistry, 281(42), 31950–31962. 10.1016/s0021-9258(19)84109-1 16920708

[IMAG.a.1192-b68] Ronchi, F., Basso, C., Preite, S., Reboldi, A., Baumjohann, D., Perlini, L., Lanzavecchia, A., & Sallusto, F. (2016). Experimental priming of encephalitogenic Th1/Th17 cells requires pertussis toxin-driven IL-1β production by myeloid cells. Nature Communications, 7(1), 11541. 10.1038/ncomms11541 PMC4873938 27189410

[IMAG.a.1192-b69] Sadeghian, M., Mastrolia, V., Rezaei Haddad, A., Mosley, A., Mullali, G., Schiza, D., Sajic, M., Hargreaves, I., Heales, S., Duchen, M. R., & Smith, K. J. (2016). Mitochondrial dysfunction is an important cause of neurological deficits in an inflammatory model of multiple sclerosis. Scientific Reports, 6(1), 33249. 10.1038/srep33249 27624721 PMC5021937

[IMAG.a.1192-b70] Schiepers, C., Van Hecke, P., Vandenberghe, R., Van Oostende, S., Dupont, P., Demaerel, P., Bormans, G., & Carton, H. (1997). Positron emission tomography, magnetic resonance imaging and proton NMR spectroscopy of white matter in multiple sclerosis. Multiple Sclerosis Journal, 3(1), 8–17. 10.1177/135245859700300102 9160342

[IMAG.a.1192-b71] Sivakolundu, D. K., Hansen, M. R., West, K. L., Wang, Y., Stanley, T., Wilson, A., McCreary, M., Turner, M. P., Pinho, M. C., Newton, B. D., Guo, X., Rypma, B., & Okuda, D. T. (2019). Three‐dimensional lesion phenotyping and physiologic characterization inform remyelination ability in multiple sclerosis. Journal of Neuroimaging, 29(5), 605–614. 10.1111/jon.12633 31148298

[IMAG.a.1192-b72] Smith, K. J., & Lassmann, H. (2002). The role of nitric oxide in multiple sclerosis. The Lancet Neurology, 1(4), 232–241. 10.1016/s1474-4422(02)00102-3 12849456

[IMAG.a.1192-b73] Soroush, A., & Dunn, J. F. (2025). A hypoxia-inflammation cycle and multiple sclerosis: Mechanisms and therapeutic implications. Current Treatment Options in Neurology, 27(1), 6. 10.1007/s11940-024-00816-4 39569339 PMC11573864

[IMAG.a.1192-b74] Srinivasan, S., & Avadhani, N. G. (2012). Cytochrome c oxidase dysfunction in oxidative stress. Free Radical Biology and Medicine, 53(6), 1252–1263. 10.1016/j.freeradbiomed.2012.07.021 22841758 PMC3436951

[IMAG.a.1192-b75] Steudler, J., Ecott, T., Ivan, D. C., Bouillet, E., Walthert, S., Berve, K., Dick, T. P., Engelhardt, B., & Locatelli, G. (2022). Autoimmune neuroinflammation triggers mitochondrial oxidation in oligodendrocytes. Glia, 70(11), 2045–2061. 10.1002/glia.24235 35762739 PMC9546135

[IMAG.a.1192-b76] Sun, X., Tanaka, M., Kondo, S., Okamoto, K., & Hirai, S. (1998). Clinical significance of reduced cerebral metabolism in multiple sclerosis: A combined PET and MRI study. Annals of Nuclear Medicine, 12, 89–94. 10.1007/bf03164835 9637279

[IMAG.a.1192-b77] Taccone, F. S., Su, F., De Deyne, C., Abdellhai, A., Pierrakos, C., He, X., Donadello, K., Dewitte, O., Vincent, J. L., & De Backer, D. (2014). Sepsis is associated with altered cerebral microcirculation and tissue hypoxia in experimental peritonitis. Critical Care Medicine, 42(2), e114–e122. 10.1097/ccm.0b013e3182a641b8 24196192

[IMAG.a.1192-b78] Tai, Y.-H., Engels, D., Locatelli, G., Emmanouilidis, I., Fecher, C., Theodorou, D., Müller, S. A., Licht-Mayer, S., Kreutzfeldt, M., Wagner, I., de Mello, N. P., Gkotzamani, S. N., Trovò, L., Kendirli, A., Aljović, A., Breckwoldt, M. O., Naumann, R., Bareyre, F. M., Perocchi, F., … Misgeld, T. (2023). Targeting the TCA cycle can ameliorate widespread axonal energy deficiency in neuroinflammatory lesions. Nature Metabolism, 5(8), 1364–1381. 10.1038/s42255-023-00838-3 PMC10447243 37430025

[IMAG.a.1192-b79] Tichauer, K. M., Hadway, J. A., Lee, T. Y., & St Lawrence, K. (2006). Measurement of cerebral oxidative metabolism with near-infrared spectroscopy: A validation study. Journal of Cerebral Blood Flow & Metabolism, 26(5), 722–730. 10.1038/sj.jcbfm.9600230 16192991

[IMAG.a.1192-b80] Trapp, B. D., & Stys, P. K. (2009). Virtual hypoxia and chronic necrosis of demyelinated axons in multiple sclerosis. The Lancet Neurology, 8(3), 280–291. 10.1016/s1474-4422(09)70043-2 19233038

[IMAG.a.1192-b81] Trapp, B. D., Vignos, M., Dudman, J., Chang, A., Fisher, E., Staugaitis, S. M., Battapady, H., Mork, S., Ontaneda, D., Jones, S. E., Fox, R. J., Chen, J., Nakamura, K., & Rudick, R. A. (2018). Cortical neuronal densities and cerebral white matter demyelination in multiple sclerosis: A retrospective study. The Lancet Neurology, 17(10), 870–884. 10.1016/s1474-4422(18)30245-x 30143361 PMC6197820

[IMAG.a.1192-b82] UCL, B. O. R. L. (2005). *Tissue spectra*. https://web.archive.org/web/20170716153131/http://www.ucl.ac.uk/medphys/research/borl/resources/intro-spectra

[IMAG.a.1192-b83] Varga, A. W., Johnson, G., Babb, J. S., Herbert, J., Grossman, R. I., & Inglese, M. (2009). White matter hemodynamic abnormalities precede sub-cortical gray matter changes in multiple sclerosis. Journal of the Neurological Sciences, 282(1–2), 28–33. 10.1016/j.jns.2008.12.036 19181347 PMC2737614

[IMAG.a.1192-b84] Vidal-Itriago, A., Radford, R. A., Aramideh, J. A., Maurel, C., Scherer, N. M., Don, E. K., Lee, A., Chung, R. S., Graeber, M. B., & Morsch, M. (2022). Microglia morphophysiological diversity and its implications for the CNS. Frontiers in Immunology, 13, 997786. 10.3389/fimmu.2022.997786 36341385 PMC9627549

[IMAG.a.1192-b85] Vitorino, R., Hojjat, S.-P., Cantrell, C. G., Feinstein, A., Zhang, L., Lee, L., O'Connor, P., Carroll, T. J., & Aviv, R. I. (2016). Regional frontal perfusion deficits in relapsing-remitting multiple sclerosis with cognitive decline. American Journal of Neuroradiology, 37(10), 1800–1807. 10.3174/ajnr.a4824 27197989 PMC5116278

[IMAG.a.1192-b86] Wakefield, A. J., More, L. J., Difford, J., & McLaughlin, J. E. (1994). Immunohistochemical study of vascular injury in acute multiple sclerosis. Journal of Clinical Pathology, 47(2), 129–133. 10.1136/jcp.47.2.129 8132826 PMC501826

[IMAG.a.1192-b87] Weaver, A., Da Silva, A. G., Nuttall, R. K., Edwards, D. R., Shapiro, S. D., Rivest, S., & Yong, V. W. (2005). An elevated matrix metalloproteinase (MMP) in an animal model of multiple sclerosis is protective by affecting Th1/Th2 polarization. The FASEB Journal, 19(12), 1668–1670. 10.1096/fj.04-2030fje 16081501

[IMAG.a.1192-b88] West, K., Sivakolundu, D., Maruthy, G., Zuppichini, M., Liu, P., Thomas, B., Spence, J., Lu, H., Okuda, D., & Rypma, B. (2020). Baseline cerebral metabolism predicts fatigue and cognition in multiple sclerosis patients. NeuroImage: Clinical, 27, 102281. 10.1016/j.nicl.2020.102281 32544855 PMC7298673

[IMAG.a.1192-b89] Witte, M. E., Geurts, J. J., de Vries, H. E., van der Valk, P., & van Horssen, J. (2010). Mitochondrial dysfunction: A potential link between neuroinflammation and neurodegeneration? Mitochondrion, 10(5), 411–418. 10.1016/j.mito.2010.05.014 20573557

[IMAG.a.1192-b90] Xie, Y., Zhang, S., Wu, D., Yao, Y., Cho, J., Lu, J., Zhu, H., Wang, Y., Zhang, Y., & Zhu, W. (2024). The changes of oxygen extraction fraction in different types of lesions in relapsing–remitting multiple sclerosis: A cross-sectional and longitudinal study. Neurological Sciences, 45(8), 3939–3949. 10.1007/s10072-024-07463-2 38492126

[IMAG.a.1192-b91] Xu, Y., Chen, Y., Zhang, X., Ma, J., Liu, Y., Cui, L., & Wang, F. (2022). Glycolysis in innate immune cells contributes to autoimmunity. Frontiers in Immunology, 13, 920029. 10.3389/fimmu.2022.920029 35844594 PMC9284233

[IMAG.a.1192-b92] Yang, R., & Dunn, J. F. (2015). Reduced cortical microvascular oxygenation in multiple sclerosis: A blinded, case-controlled study using a novel quantitative near-infrared spectroscopy method. Scientific Reports, 5(1), 16477. 10.1038/srep16477 26563581 PMC4643232

[IMAG.a.1192-b93] Yang, R., & Dunn, J. F. (2019). Multiple sclerosis disease progression: Contributions from a hypoxia–inflammation cycle. Multiple Sclerosis Journal, 25(13), 1715–1718. 10.1177/1352458518791683 30052113 PMC6826859

[IMAG.a.1192-b94] Zhang, Q., Srinivasan, S., Wu, Y., Natah, S., & Dunn, J. F. (2010). A near-infrared calibration method suitable for quantification of broadband data in humans. Journal of Neuroscience Methods, 188(2), 181–186. 10.1016/j.jneumeth.2010.01.037 20156483

[IMAG.a.1192-b95] Zhao, C., Coons, S., Cui, M., Shi, F.-D., Vollmer, T., Ma, C., Kuniyoshi, S. M., & Shi, J. (2008). A new EAE model of brain demyelination induced by intracerebroventricular pertussis toxin. Biochemical and Biophysical Research Communications, 370(1), 16–21. 10.1016/j.bbrc.2008.02.161 18339308

[IMAG.a.1192-b96] Zou, M., Feng, Y., Xiu, Y., Li, Y., Zhang, Y., Fan, J., Li, H., Cao, J., He, W., & Jin, W.-N. (2022). Pertussis toxin–induced inflammatory response exacerbates intracerebral haemorrhage and ischaemic stroke in mice. Stroke and Vascular Neurology, 7(1), 29–37. 10.1136/svn-2021-000987 34341068 PMC8899681

